# Transcriptomics reveal potential vaccine antigens and a drastic increase of upregulated genes during *Theileria parva* development from arthropod to bovine infective stages

**DOI:** 10.1371/journal.pone.0204047

**Published:** 2018-10-10

**Authors:** Triza Tonui, Pilar Corredor-Moreno, Esther Kanduma, Joyce Njuguna, Moses N. Njahira, Steven G. Nyanjom, Joana C. Silva, Appolinaire Djikeng, Roger Pelle

**Affiliations:** 1 Biosciences eastern and central Africa—International Livestock Research Institute (BecA-ILRI) Hub, Nairobi, Kenya; 2 Department of Biochemistry, Jomo Kenyatta University of Agriculture and Technology, City Square, Nairobi, Kenya; 3 John Innes Centre, Norwich Research Park, Colney Lane, Norwich, United Kingdom; 4 Department of Biochemistry, University of Nairobi, Nairobi, Kenya; 5 International Centre of Insect Physiology and Ecology (ICIPE), Nairobi, Kenya; 6 Institute for Genome Sciences and Department of Microbiology and Immunology, University of Maryland School of Medicine, Baltimore, United States of America; 7 Centre for Tropical Livestock Genetics and Health, The Roslin Institute and Royal (Dick) School of Veterinary Studies, The University of Edinburgh, Scotland, United Kingdom; Institut national de la santé et de la recherche médicale - Institut Cochin, FRANCE

## Abstract

*Theileria parva* is a protozoan parasite transmitted by the brown ear tick *Rhipicephalus appendiculatus* that causes East Coast fever (ECF) in cattle, resulting in substantial economic losses in the regions of southern, eastern and central Africa. The schizont form of the parasite transforms the bovine host lymphocytes into actively proliferating cancer-like cells. However, how *T*. *parva* causes bovine host cells to proliferate and maintain a cancerous phenotype following infection is still poorly understood. On the other hand, current efforts to develop improved vaccines have identified only a few candidate antigens. In the present paper, we report the first comparative transcriptomic analysis throughout the course of *T*. *parva* infection. We observed that the development of sporoblast into sporozoite and then the establishment in the host cells as schizont is accompanied by a drastic increase of upregulated genes in the schizont stage of the parasite. In contrast, the ten highest gene expression values occurred in the arthropod vector stages. A comparative analysis showed that 2845 genes were upregulated in both sporozoite and schizont stages compared to the sporoblast. In addition, 647 were upregulated only in the sporozoite whereas 310 were only upregulated in the schizont. We detected low p67 expression in the schizont stage, an unexpected finding considering that p67 has been reported as a sporozoite stage-specific gene. In contrast, we found that transcription of p67 was 20 times higher in the sporoblast than in the sporozoite. Using the expression profiles of recently identified candidate vaccine antigens as a benchmark for selection for novel potential vaccine candidates, we identified three genes with expression similar to p67 and several other genes similar to Tp1—Tp10 schizont vaccine antigens. We propose that the antigenicity or chemotherapeutic potential of this panel of new candidate antigens be further investigated. Structural comparisons of the transcripts generated here with the existing gene models for the respective loci revealed indels. Our findings can be used to improve the structural annotation of the *T*. *parva* genome, and the identification of alternatively spliced transcripts.

## Introduction

*Theileria parva* is an obligate intracellular protozoan parasite transmitted by the ixodid tick *Rhipicephalus appendiculatus*, commonly known as ‘brown ear tick’ [1]. The parasite causes East Coast fever (ECF), a lymphoproliferative disease of cattle endemic in the regions of eastern, southern and central Africa [2,3]. ECF accounts for over 1 million cattle deaths annually and over USD 300 million in terms of economic losses per year [4]. The life cycle of *Theileria parva* alternates between the cattle and the tick. The tick gut harbors the sexual stage of the parasite life cycle following ingestion of piroplasms in the red blood cells of an infected bovine host. Final parasite differentiation occurs in the immature stages of the tick once they have detached from the host. A club-shaped motile kinete forms within the zygote, as the tick molts, is then released into the body cavity and then migrates into the salivary glands via the hemolymph. The kinetes then invade the epithelial cells of the tick salivary glands and develop into a large syncytium, called sporoblast, within which several thousand minute, elongated sporozoites originate, during the early part of a tick’s next feeding. As the tick feeds, sporozoites are released into the saliva and inoculated into the skin of the mammalian host (feeding site). Sporozoites then invade host lymphocytes and differentiate into multinucleate bodies, termed as schizonts, in the lymphocyte cytoplasm after a period of three days. The schizonts cause transformation of the infected host cells thereby inducing a cancer-like phenotype [5]. The parasite establishes itself within the fundamental components of the host cell because it adequately evades lysosomal digestion and controls the host cell’s proliferation machinery [6]. Some of the schizonts undergo merogony, giving rise to merozoites. These merozoites mature into piroplasms in the red blood cells and are infective to ticks. It is the tick larvae and nymph that ingest the parasite by feeding on an infected bovine host, and transmit the parasites as the next tick instar. Upon infection, *T*. *parva*-transformed cells depend neither on exogenous growth factors nor on antigenic stimulation to proliferate. Rather, they express a broad range of cytokines and lymphokines that play a pivotal role in contributing to cell proliferation and subsequent survival of parasitized cells [7]. What drives host cells to proliferate and maintain a cancerous phenotype following infection by *T*. *parva* is unknown. However, the dependence of the transformed phenotype on the presence of the parasite is well established, because when the schizont is killed with drugs, such as parvaquone, buparvaquone and compound BW720c [1], the host cell reverts to normal growth characteristics. This suggests that the parasite does not cause permanent changes to the host genome but rather usurps pathways controlling the cell cycle of the infected cell. In addition, the severity of ECF is generally dependent on the level of parasitemia whereas the schizont stage of infection represents the main pathogenic state of the disease [8]. Understanding the profile of gene expression in the parasite in the early stages of sporozoite infection that lead to transformation of lymphocytes by schizonts could shed light on the genes that are responsible for establishment of the disease and may represent potential targets for vaccine and diagnostic development. Therefore, in the present study, we have carried out comparative transcriptomic analyses of *T*. *parva* from the sporoblast and sporozoite life cycle stages in the tick, as well as the schizont stage in the bovine host. This was done using the Illumina MiSeq sequencing platform and sequences generated were processed and analyzed using bioinformatic tools and expression profile of known ECF candidate vaccine antigens.

## Materials and methods

### Sample collection and RNA purification

**Fig 1:**
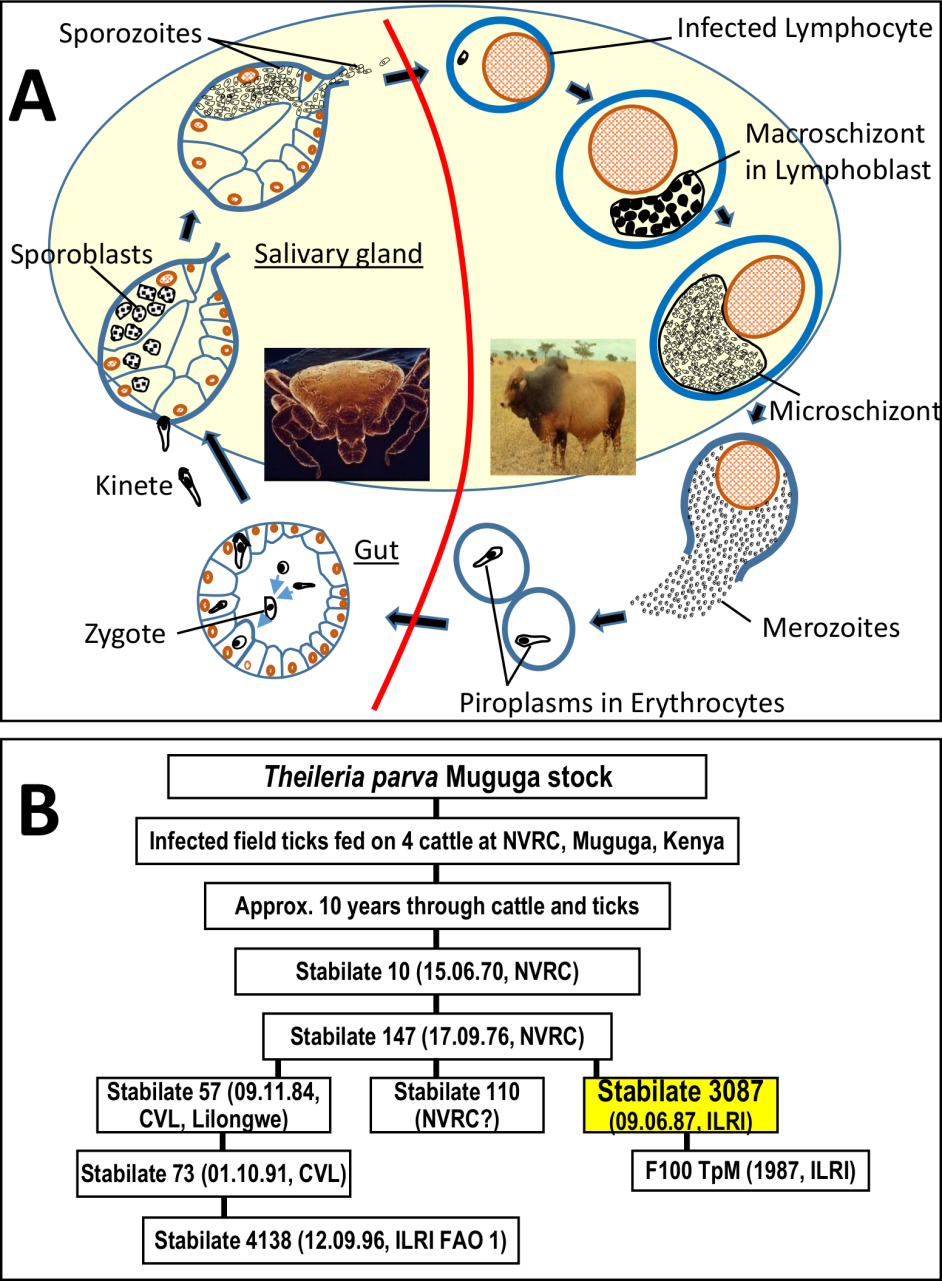
**A) *Theileria parva* life cycle stages** (not drawn to scale). The tick and cattle stages (sporoblast, sporozoite and schizont) shadowed are the ones reported in the present study. The red line separates the bovine from the tick vector stages. **B) Flow chart of the origin of the stabilate *T*. *parva* Muguga 3087 (stabilate 3087) used in this study (shadowed box)**. The cow F100 was infected with TpM 3087 to generate F100 TpM. The stabilate 4138 is the Muguga component of three stock components of the Muguga Cocktail ITM live vaccine. The date (day, month and year) and place of preparation are indicated where known. NVRC; National Veterinary Research Centre, Muguga, Kenya. CVL; Central Veterinary Laboratory, Lilongwe, Malawi.

*Rhipicephalus appendiculatus* tick salivary glands infected with *Theileria parva* Muguga stabilate 3087 [9] for the purification of sporoblast and sporozoite forms of the parasite (Fig 1A) were supplied by the tick vector unit of the International Livestock Research Institute (ILRI). The stabilate 3087 was prepared as follows: the Muguga stock was isolated by feeding field ticks from Muguga, in Kenya, on four cattle at the National Veterinary Research Centre (NVRC), Muguga, and, after several tick-cattle passages, the stabilate number 10 was made in 1970. From it, NVRC made stabilate 147, which was used by ILRI (then ILRAD) to prepare stabilate 3087 [10]. Fig 1B is a flow chart outlining the origin of the TpM 3087.

For the sporoblast form, dissected salivary glands from 2-day fed infected adult ticks were used whereas for the sporozoite form, tick salivary grands from 4-day fed infected adult ticks were used. So, about 350 tick salivary glands (2000 acini/salivary gland with averages of about 50 infected acini per tick and 30,000 sporozoites per acinus) were aseptically dissected from infected adult ticks and collected in 2 ml RPMI 1640 (Flow Laboratories) containing 5% foetal calf serum on ice then ground manually in a sterile chilled sintered glass tissue grinder by a gentle up, down and circular motion of the pestle until a uniform cloudy suspension is formed. The homogenate was centrifuged at 300 x g for 5 minutes in a pre-chilled centrifuge at 4°C to remove large tissue debris pelleted at the bottom of the tube. The supernatant containing the sporozoites and sporoblast, respectively, were loaded onto a column of an 8 ml DEAE 52 cellulose resin in the sporozoite buffer (per 1 litre: 9.4 g NaCl, 9 g D-glucose, 4.16 g Na_2_HPO_4_, 9 g Tris base, pH 8.2) packed in a 10 ml polypropylene syringe containing a Whatman 4 qualitative filter paper (Cat. # 1004–150) to hold the resin. Then 4 ml fractions of sporozoite buffer were applied to the column and the sporozoites or sporoblasts were collected in 3.5 ml fractions while ensuring that the DEAE-cellulose bed does not run dry at any one point before and during collection. The sporozoites/sporoblasts fractions were pooled (~10 ml) into chilled falcon tubes and centrifuged at 12,000 x g for 10 minutes at 4°C. The supernatant was carefully pipetted off, and the remaining small yellowish/white pellet of parasites was stored at -80°C for RNA extraction. The schizont form of the parasite (Fig 1) was purified from approx. 2 x 10^8^ cells obtained from the *in vitro* established *T*. *parva* (Muguga) schizont-infected bovine peripheral blood mononuclear cell line TpM 3087 at the ILRI tissue culture unit as described [11]. Samples from the three parasite stages (i.e., sporoblast, sporozoite and schizont) were processed for total RNA purification using the RNAzol^®^ RT isolation kit according to the manufacturers’ instructions (Sigma-Aldrich). Purified RNA was quantified by Nanodrop^®^-1000 spectrophotometer (Nanodrop technologies, Delaware, USA). The integrity of RNA was verified using 1.5% agarose RNA gel as described by Pelle and Murphy [12]. Poly(A)^+^ RNA was purified from the total RNA isolated from the three stages of the parasite, using FastTrack® MAG beads kit according to the manufacturer’s instructions. The integrity of RNA-poly (A^+^) was checked on 1.5% agarose gel electrophoresis.

### cDNA library preparation

#### Normalization

Normalization of the isolated mRNA from the sporoblast, sporozoite and schizont stages of the parasite was done using Ambion® ERCC Spike-In Control. Briefly, 2 μl of ERCC spike in mix was added to 8 μl (50 ηg) of mRNA sample from each of the three stages of the parasite. The importance of adding this internal control in gene expression profiling study was to eliminate variations that occur due of such factors as quality of the starting material, RNA yield, platform used, and human error during the experiment, among others. Ambion® ERCC Spike-In Control is composed of a set of unlabelled poly-adenylated transcripts that mimic natural eukaryotic mRNAs, added to the RNA analysis to measure against performance criteria of the platform used.

#### Truseq library construction

The 10 μl purified mRNA spiked with Ambion® ERCC RNA control were then used to prepare each library using the Truseq stranded Illumina protocol according to the manufacturer’s instructions. The concentrations of the libraries were checked using Qubit (Fisher Scientific) broad range and high sensitivity parameters. The integrity of the library fragments was checked using Agilent Bioanalyzer and 1.2% agarose gel electrophoresis with TAE buffer. The Qubit results >30.0 ηg/μl were considered of high concentrations and diluted to bring the concentrations of the samples to approximately 15 ηg/μl to avoid over-clustering errors on the sequencer. The libraries were then prepared for sequencing on an Illumina MiSeq sequencer according to the manufacturer’s instructions. The reads obtained were then exported to the ILRI high performance cluster (HPC) server for data analysis.

### Sequence data filtering

MiSeq sequencing reads were cleaned using fastqc and fastx trimmer tool kit. Quality checks were performed on the raw read using fastqc/0.11.3. Then the adapters were clipped from the fasta sequences using fastx trimmer/0.0.13. Dynamic trimming of the end terminal reads was performed using SolexaQA++/3.1.3 and the complementary reads were paired using python/3.4.3.

### Transcript quantification and differential expression analysis

The transcript abundances in the RNA-Seq samples were quantified using Kallisto version 0.43.0 [13] (https://pachterlab.github.io/kallisto/about.html). Kallisto performs a pseudo-alignment using the reference transcriptome to determine the compatibility of reads with target transcripts. After indexing the *Theileria parva* reference transcriptome (Accession no: GCF_000165365.1_ASM16536v1), the paired-end reads previously trimmed were mapped to the indexed transcriptome. Comparison was done using both the original annotation released with the original 2005 genome [14] and the updated genome annotation, a manual curation based on current evidence, including schizont RNA (Tretina et al., in preparation; http://igs-ilri.igs.umaryland.edu/eukaryotic.php). In case of discrepancies relative to our transcripts’ sequence information, the version of annotation used is stated. The locus tag identifiers for the new genome annotation are very similar to those in the original annotation for genes with no or minor structural changes. For instance, TP01_0001 simply becomes TpMuguga_01g00001. However, in the case of genes with a fundamentally difference structure, the gene numbering will be altered to start in the 2000’s. The Sleuth R package (http://pachterlab.github.io/sleuth/) [15] was used to identify differentially expressed genes at the different infection stages. Sleuth was used to explore previously calculated Kallisto quantifications and bootstraps; and read counts were normalized to Transcripts Per Kilobase Million (TPM) thus accounting for gene length and sequencing depth. Genes were considered differentially expressed when q.value cut off (FDR adjusted p-value using Benjamini-Hochberg model [16]) was lower than 0.01. Paired reads were also mapped to the *T*. *parva* genome using TopHat2 version 2.1.0 [17] and Bowtie2 version 2.2.8. Running options for TopHat2 included a maximum of 2 alignments for one read (2 maximum multihits, -g 2), 10 mismatches (-N 10), 2 splice mismatches (-m 10), 10 bp gaps (—read-gap-length 10) and an edit distance of 10 (—read-edit-dist 10). Cufflinks version 2.2.1 [18] was used to estimate transcript abundances and analyse differences in expression by isoform. The R package Cluster [19] was used to find genes with similar expression to the known *T*. *parva* antigens.

### Unmapped Theileria parva reads

Kallisto was used to obtain the reads that did not map the reference transcriptome. These reads for all the stages were subjected to sequence similarity searches against the National Center for Biotechnology Information (NCBI) non-redundant (nr) database using the BLASTN algorithm with an E-value of 10^−10^. Only the best hit with a 100% identity between query and subject was retrieved for each unmapped read. Unmapped reads blasting the *Theileria parva* genome were further considered for investigation. Genes that appeared repeatedly in the different stages were counted for each infection stage and subjected to a count-based differential expression analysis using DESeq2 [20].

### Functional enrichment of differentially expressed genes

Functional annotation of the significantly differentially expressed genes was assessed using Database for Annotation, Visualization and Integrated Discovery (DAVID) (NIAID and NIH). Gene ontology (GO) term enrichment was analysed individually for functional classification of the upregulated genes in each of the three infection stages.

*In silico* search of N-terminal signal peptide, nuclear localization signal, transmembrane domain and C-terminal GPI anchor signal as well as prediction of protein structure and function and non-classical protein secretion were done using SignalP 4.1 [21], Protter [22], PredictProtein [23], PredGPI [24], PredictProtein server [25] and SecretomeP 2.0 server [26], respectively.

## Results

### Kallisto improves mapping to the reference transcriptome in the three infection stages, relative to alternative

**Fig 2:**
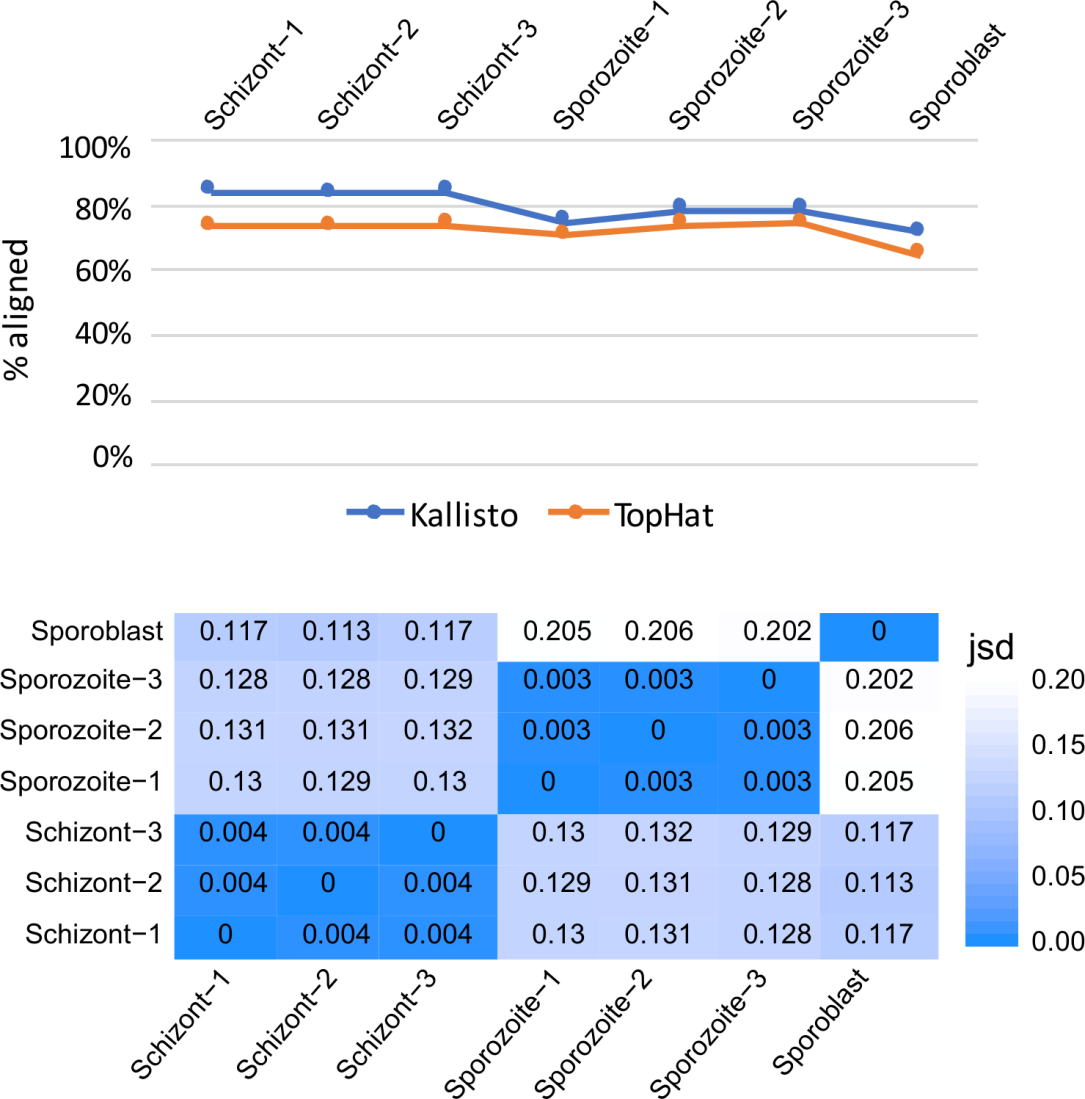
Kallisto and TopHat2 comparison and similarity between samples. **A**) Percentage of paired-end reads mapping to the reference transcriptome using Kallisto (blue line) and to the reference genome using TopHat2/Bowtie2 (orange line). **B**) Jensen-Shannon divergence (JSD) heatmap between each pair of samples.

The high cost associated with the preparation of the sporoblast form of the parasite prevented the generation of more than one sample for this life cycle stage; in contrast, three replicates were obtained for each the schizont and sporozoite stages. Transcript abundance in each replicate was quantified using Kallisto. The average percentage of mapped reads for all the replicates was 79.4%. The mapping percentage obtained using TopHat2 was 72.1%. We decided to proceed using the Kallisto output for the following steps of the differential expression analysis, as the mapping success rate was higher for all the replicates (Table 1, Fig 2). The percentage of paired-end reads mapped was highest in the schizont stage and lowest in the sporoblast.

**Table 1:** Percentage of reads mapped to reference transcriptome using Kallisto and to the reference genome using TopHat2 after trimming.

Sample	Reads processed after trimming	Reads mapped using Kallisto	Fraction mapped with Kallisto	Reads mapped using TopHat2	Fraction mapped with TopHat2
**Sporoblast**	1914069	1370307	71.59%	1242257	64.60%
**Sporozoite 3**	1754486	1380145	78.66%	1303559	74.30%
**Sporozoite 2**	1956212	1534430	78.44%	1450258	74.10%
**Sporozoite 1**	2119830	1587160	74.87%	1501601	70.80%
**Schizont 3**	1989897	1677204	84.29%	1469898	73.80%
**Schizont 2**	2214754	1855253	83.77%	1628811	73.50%
**Schizont 1**	1952716	1647373	84.36%	1436313	73.50%

To assess the similarity between replicates in the sporozoite and the schizont phases, we used Sleuth to build a heatmap showing the Jensen-Shannon divergence between pairs of samples. There was no significant difference among replicates of the same life cycle stage (Fig 2B). The largest distances (0.202–0.206) were observed between the sporoblast and sporozoite stages.

Overall, using both the original and the re-annotated genomes, transcripts of 3924 genes encoded in the nuclear genome could be detected, which had good level (TPM>2) of Transcripts Per Kilobase Million (TPM) in at least one of the three stages examined (Supplementary S1 Table).

### Unmapped reads mainly originate from mammalian and tick genomes, and some from *Theileria parva*

**Fig 3:**
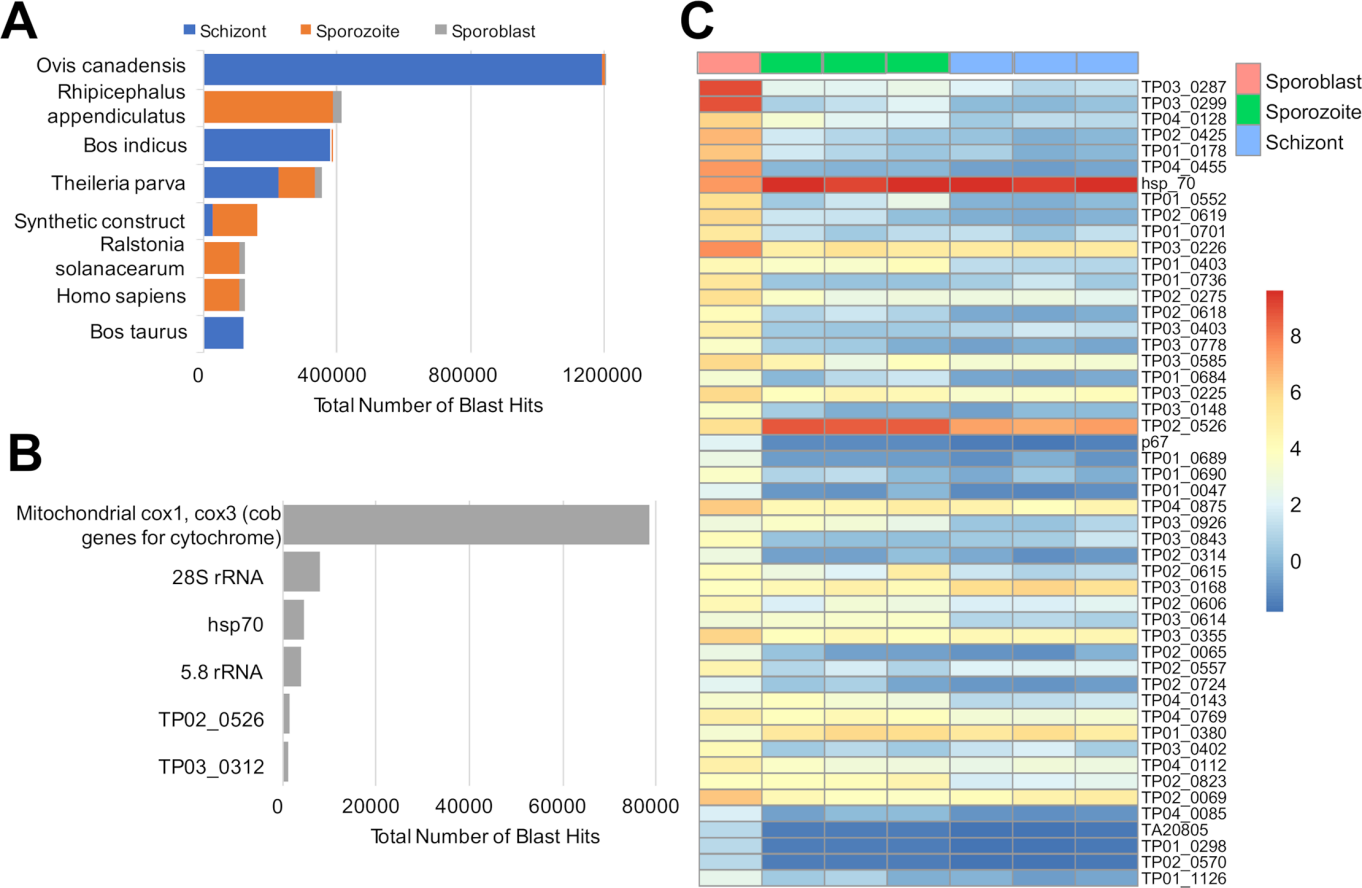
Analysis of unmapped reads. **A**) Count (>1000 showed only) of the Blast hits from unmapped reads after using Kallisto for the three infection stages. **B**) Count of Blast hits to *T*. *parva* genome (only targets with >1000 showed only). **C**) Heatmap showing differentially expressed genes (p-adj < 0.05) obtained from the unmapped reads for each infection stage.

For each sample, reads that remained unmapped after running Kallisto were subjected to sequence similarity searches. The best hit for each read was retrieved. The species with over 1,000 hits in the BLAST output (89.61% of the total hits) were plotted together to find their prevalence in the RNA-seq samples. Most of the reads with matches originated from schizont and sporozoite samples, while there were not many hits from originally unmapped reads from sporoblast sample. This is due, in part, to the presence of a single sporoblast sample compared to three for the other two stages, but also to differences in the quality and completeness of the tick and the bovine genomes. The more preliminary status of the *R*. *appendiculatus* genome will prevent the identification of homologous regions for some reads of tick origin. In total, 41.41% of the unmapped reads, mostly from the three schizont replicates, hit the *Ovis canadensis* genome, but we can confidently assume that those genes are orthologues to the bovine host genome (*Bos indicus*) (Fig 3A). 13.49% of the unmapped reads, mainly from the sporozoite replicates, mapped to *Rhipicephalus appendiculatus* (the tick vector) genome. Close to 20% of the reads, mostly from schizont samples, mapped to the bovine host genome (*Bos indicus*) or to *Bos taurus*, and undoubtedly originated from the bovine host’s genome. Other common blast hits included synthetic constructs probably coming from sequencing, as well as from *Ralstonia solanacearum* (a Gram-negative soil-borne plant pathogenic bacterium). The fact that hits to the latter are only present in the tick stages suggests that ticks spread this devastating phytopathogenic bacteria, or a closely related species. Finally, 11.19% of the reads hit the *Theileria parva* genome. The latter were considered for further investigation.

Out of 267,117 total hits to *T*. *parva*, almost 30% corresponded to mitochondrial genes (cytochrome oxidase) (Fig 3B); this is not unexpected, since the transcriptome to which the data was mapped included only transcripts from genes encoded in the four *T*. *parva* nuclear chromosomes. We did not observe any significant difference in the expression of these genes among the three infection stages (Fig 3C). Interestingly, a significant number of reads mapped to the nuclear chromosomes of *T*. *parva* (Supplementary S1 Table). The most likely explanation is that they were incorrectly annotated and hence incomplete in the transcriptome file used as reference for read mapping, which prevented the reads to map. We could observe differences in expression in some of these genes, which appear to be more highly expressed during the sporoblast stage. Examples include TP01_0736, TP03_0287, TP03_0299 and TP04_0455 (2258, 17464.5, 11757.8 and 1636.7 reads, respectively). Inspection of the schizont RNAseq data used in the re-annotation suggest that the first three may be missing 5’ coding or non-coding exons, but the very low expression levels (2 < reads < 80) make acurrate gene structure inference difficult. In contrast, we found a few unmapped genes highly expressed in schizont and/or sporozoite while the expression is lowest in sporoblast, such as TP02_0526 and TP02_0148, which encodes HSP_70. The high level of gene expression in these two genes (average read counts in the schizont RNAseq dataset was 220 and 214, respectively) shows that the current gene structure is favoured by the vast majority of the reads, but that some reads may be present in support of (possibly stochasticly) alternatively spliced trasncripts. Differentially expressed genes (p-adj < 0.05) can be found in the supplementary S1 File.

### Massive increase in the number of differentially expressed genes during schizont stage

**Fig 4:**
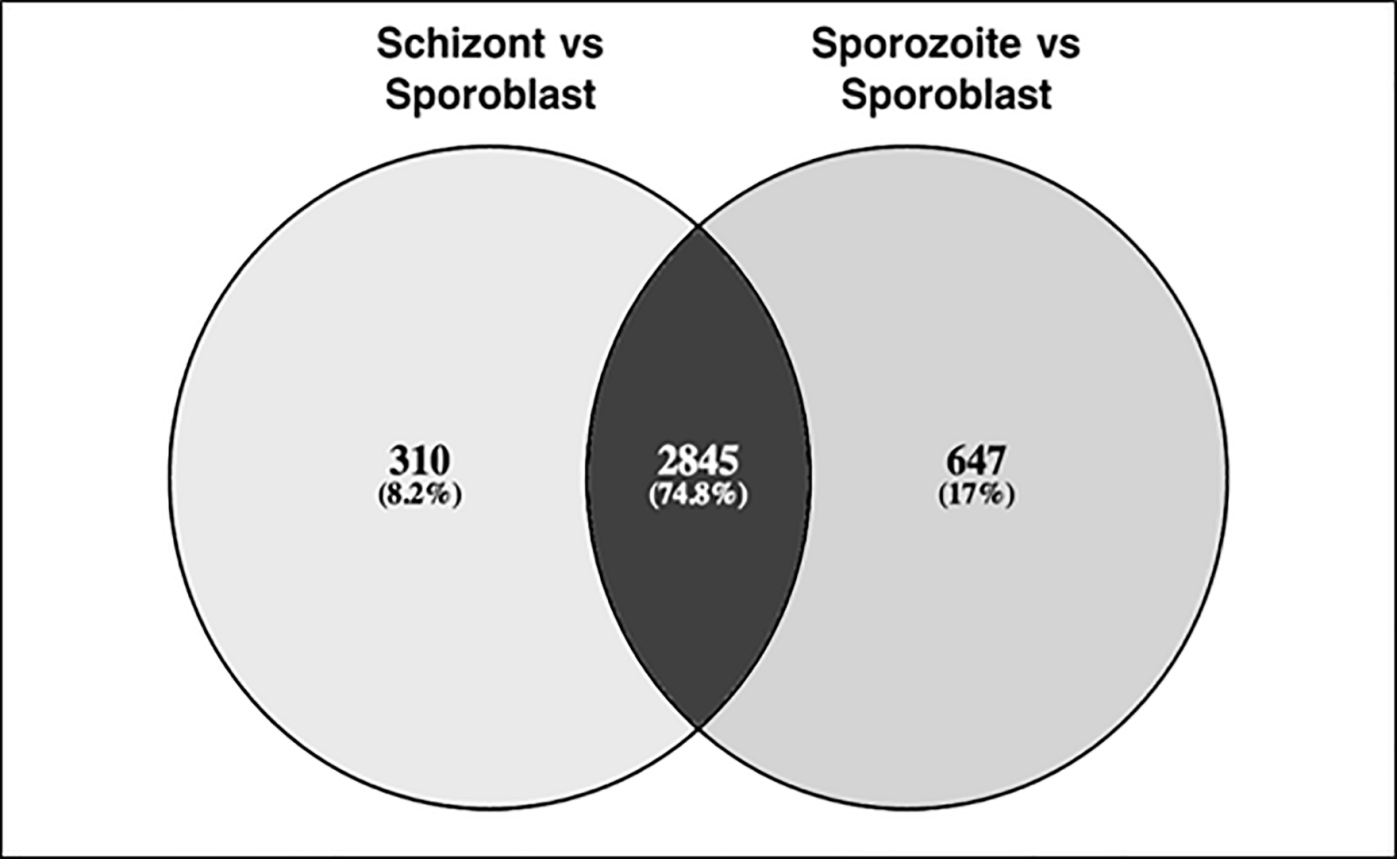
A pairwise comparison of sporoblast against sporozoite and schizont stages. 2845 genes differentially expressed in sporozoite and schizont stages in comparison to sporoblast, plus 647 and 310 only differentially expressed relative to the sporozoite or the schizont stages, respectively.

Gene expression, measured in TPM, was estimated for all genes, and differentially expressed genes between stages were identified (Supplementary S2 Table). Pairwise comparisons were performed between sporoblast and each of the other two stages (sporozoite and schizont). 2845 genes were differentially expressed (fold change up to +8) in both comparisons (Fig 4). In addition, 647 were only differentially expressed relative to the sporozoite stage, and 310 were only differentially expressed relative to the schizont stage.

**Fig 5:**
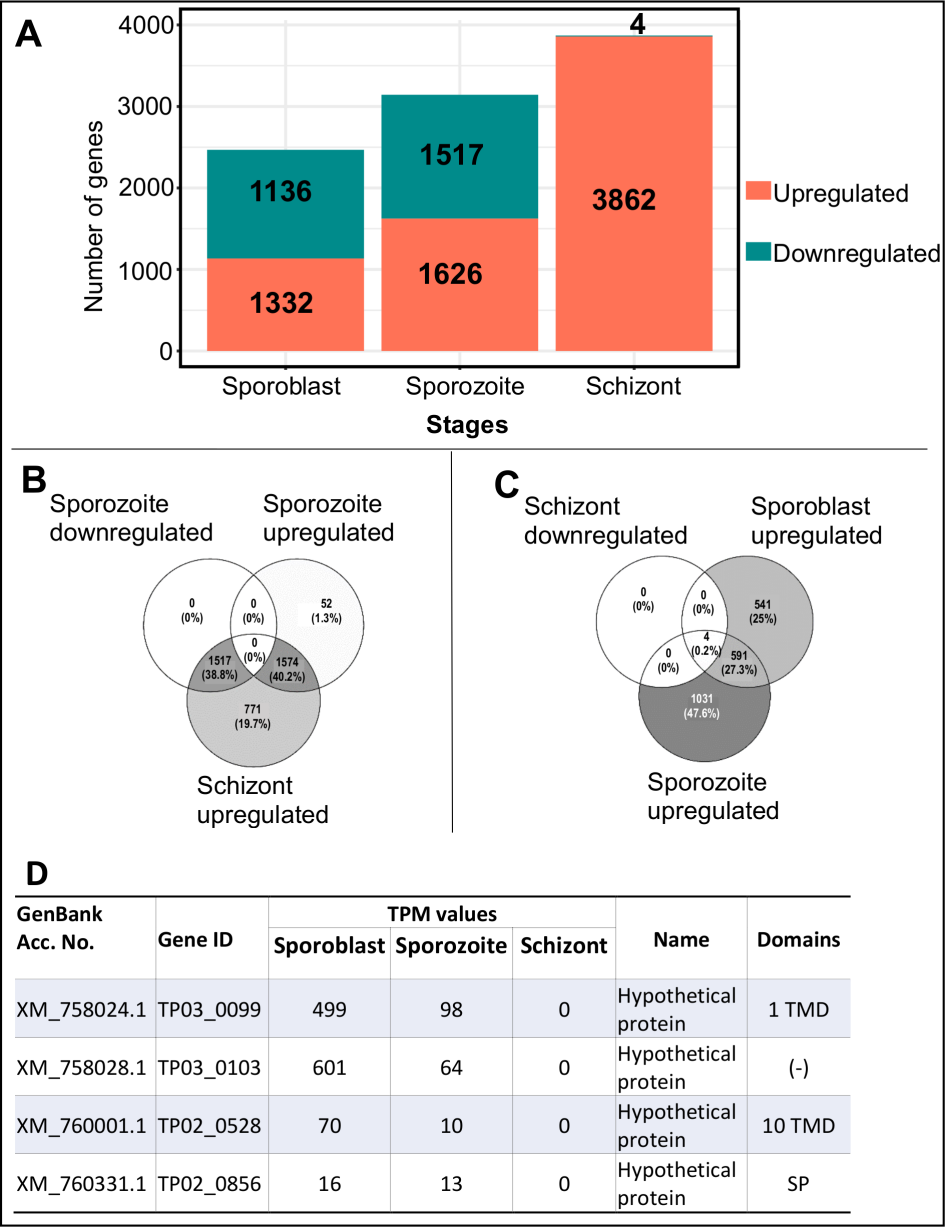
Distribution of differentially expressed genes (p-adj < 0.01). **A)**. Total number of genes upregulated (coral) and downregulated (green) in the three life cycle stages of the parasite. **B)** Venn diagram comparing the upregulated genes in sporozoite and schizont to the downregulated genes in sporozoite. **C)** Venn diagram comparing the upregulated genes in sporoblast and sporozoite to the downregulated genes in schizont. **D)** List of four downregulated genes in the schizont stage of the parasite. TMD, transmembrane domain; SP, signal peptide; (-), none.

The total number of upregulated and downregulated genes in each individual stage (in comparison to the other two stages) is represented in Fig 5. All the upregulated and downregulated genes for each stage can be found in the S1 Table. The number of differentially expressed genes increased with the infection progression. For the two first stages (sporoblast and sporozoite) the number of up- and down-regulated genes was close to 50% of all differentially expressed loci, in contrast to the schizont stage, where over 3800 genes were upregulated in comparison to the other stages (the last Ensembl transcriptome included 4079 protein-coding genes). In this last stage (schizont), only four genes (TP02_0528, TP02_0856, TP03_0099 and TP03_0103) were considered downregulated (with 0 TPM value in schizont). However, in the schizont the level of expression was moderate with the highest expressed gene having only 4621 TPM (TP01_1228) compared to 17,464 TPM and 11,151 TPM for the sporoblast (TP03_0287) and sporozoite (TP04_0437) stages, respectively. Some genes were expressed only in two stages whereas the top 10 most highly expressed genes were all in the tick stages (Table 2).

**Table 2:** The top 10 most highly expressed genes.

GenBank Acc. No.	Locus Tag / 2005	Locus Tag ID^a^	Average TPM			Product name	Domain
			Splast	Spzoit	Schiz		
XM_758212.1	TP03_0287	TpMuguga_03g00287	17464	840	21	p67 antigen	SP, 1 TMD
XM_758077.1	TP03_0152	TpMuguga_03g00152	15623	8710	3212	Histone H3	(-)
XM_758613.1	TP04_0071	TpMuguga_04g00071	12948	2795	1695	Histone H2B	(-)
XM_758224.1	TP03_0299	TpMuguga_03g00299	11757	413	3	Hypothetical protein	SP
XM_760084.1	TP02_0611	TpMuguga_02g00611	9103	1848	1798	Histone H2A	(-)
XM_761498.1	TP01_1070	TpMuguga_01g01070	8512	2152	2226	Ubiquitin/ ribosomal fusion protein	(-)
XM_758979.1	TP04_0437	TpMuguga_04g00437	15	11151	539	p104 antigen	SP, GPI
XM_760968.1	TP01_0541	TpMuguga_01g00541	0	9630	5	Hypothetical protein	(-)
XM_759182.1	TP04_0640	TpMuguga_04g00640	136	8957	671	Hypothetical protein	2 TMD
XM_758118.1	TP03_0193	TpMuguga_03g00193	45	8806	10	Hypothetical protein	(-)

^a^Locus Tag Identifiers according to the updated whole genome gene structural re-annotation (Tretina et al., in preparation).

Splast, sporoblast; Spzoit, sporozoite; Schiz, schizont. GPI, GPI anchor; SP, signal peptide; TMD, transmembrane domain; (-), none.

We examined the overlap between genes in the different stages using Venn diagrams. We observed that 1574 genes were upregulated in both the sporozoite and the schizont stages. All the genes that were downregulated in sporozoite showed an increased expression in the schizont, except the four hypothetical genes described above that were not expressed in schizont (0 TPM) and were most abundant in the sporoblast (Fig 5D).

### Function potential of the four hypothetical proteins within the top 10 hits

Four of the ten most highly expressed genes coded for hypothetical proteins with unknown function. We conducted further sequence and motif sequence similarity analyses to attempt to generate new hypothesis regarding their possible functions. The results are described below.

**TpMuguga_04g00640** is considerably longer than **TP04_0640** (in the original 2005 genome annotation), with one extra exon where an intron was previously annotated, and a much longer last (4^th^) exon. It now encodes a small protein of 114 amino acid residues (compared to 50 residues in TP04_0640), without a signal peptide but with two putative transmembrane domains. Furthermore, this protein contains two casein kinase II phosphorylation sites (SMID and STVE) located in its intracellular region. BLASTX search revealed a significant homology with the fission yeast endoplasmic reticulum membrane meiotically up-regulated gene 84 protein, MUG84_SCHPO (BLAST expectation value 6e-21), which plays a role in the meiotic cell cycle. TpMuguga_04g00640 showed strong homology to proteins in other piroplasms, including *Theileria*, *Babesia* and *Cytauxzoon* species.

**TpMuguga_03g00299** has similar structure to the original protein annotated in this genomic region, **TP03_0299.** It encodes a hypothetical protein of 770 amino acids containing a signal peptide, which suggests that it is released in the host cytosol. In contrast, the absence of a classical nuclear localization signal suggests that this protein is not directed to the host nucleus but may either be confined in the host cytoplasm or could be processed through ER/Golgi-dependent secretion pathways where it would be processed and presented on the surface of the infected host cell as an antigen epitope in the context of the major histocompatibility complex (MHC) class I. We also assessed whether this protein had other known motifs and identified several potential post-translational modification sites including one N-glycosylation (NSTD), one cyclic cAMP- and cGMP-dependent protein kinase phosphorylation (KRKT), seven protein kinase C phosphorylation (TSK, TPR, TRK, TDR, SER, SEK and SSR), five casein kinase II phosphorylation sites (SPLD, SESD, TSKD, SNED and STGE), and eleven N-myristoylation (GSGIGI, GNSDNF, GVTVTQ, GVTVTQ, GTTPSV, GAIASS, GTTAAS, GLGMGG, GTGAAS, GVSVGT and GIMLGE) sites as well as one cell attachment sequence (RGD). An ortholog has been detected in *T*. *annulata* only.

**TpMuguga_01g00541** is identical to **TP01_0541.** It encodes a protein of 209 amino acid residues in length with neither a classical signal peptide of secreted proteins nor a transmembrane domain or a GPI anchor motif of surface proteins. SecretomeP 2.0 software predicted NN-score of 0.57 for TP01_0541 protein. Using the PredictProtein program, we found that the TP01_0541 protein contains two potential disulfide bonds from the cysteine residues 17/33 and 115/164, respectively. It also contains two N-glycosylation (NYSY and NETE), two protein kinase C phosphorylation (SLK and TRK), seven casein kinase II phosphorylation (SLVE, SALE, TNFD, SEGE, SLSD, SQED and TIED), one tyrosine kinase phosphorylation (KEFDNENY) and one N-myristoylation (GSLKSL) sites, suggesting its involvement in numerous protein-protein activities. In addition, this protein also contains a leucine zipper pattern (LYDYGTSLVEYYRCLFQLYFNL). Most interestingly, no orthologs have been identified in other piroplasm genomes, and our updated tblastn searches against all Piroplasma genomes in EuPathDB confirm this result, having returned no matches other than in *T*. *parva*.

**TpMuguga_03g00193** is again identical to **TP03_0193.** It encodes a 346 amino acid-long protein. Much like the TP01_0541-encoded protein, this protein also contains no signal peptide, transmembrane domains or a GPI anchor motif. It contains several potential post-translational modification motifs including three N-glycosylation (NNSS, NVTD and NVTM), one cAMP- and cGMP-dependent protein kinase phosphorylation (RRLT), eleven protein kinase C phosphorylation (TKK, TRK, STK, SQK, THK, TEK, SLK, SQR, TLR, TTK and SKK), five casein kinase II phosphorylation (TKKD, SEQE, TYGD, TYED and TTKD), one tyrosine kinase phosphorylation (RTLERRY) and three N-myristoylation (GLHISQ, GCERCW and GTSITK) sites. Also of interest is the presence on a non-ordinary secondary structure (NORS) spanning amino acids 1 to 90. An ortholog is present in *T*. *annulata* but not in other piroplasms.

### Functional annotation of upregulated genes reveals activation of plasma membrane components and translation signals

**Fig 6:**
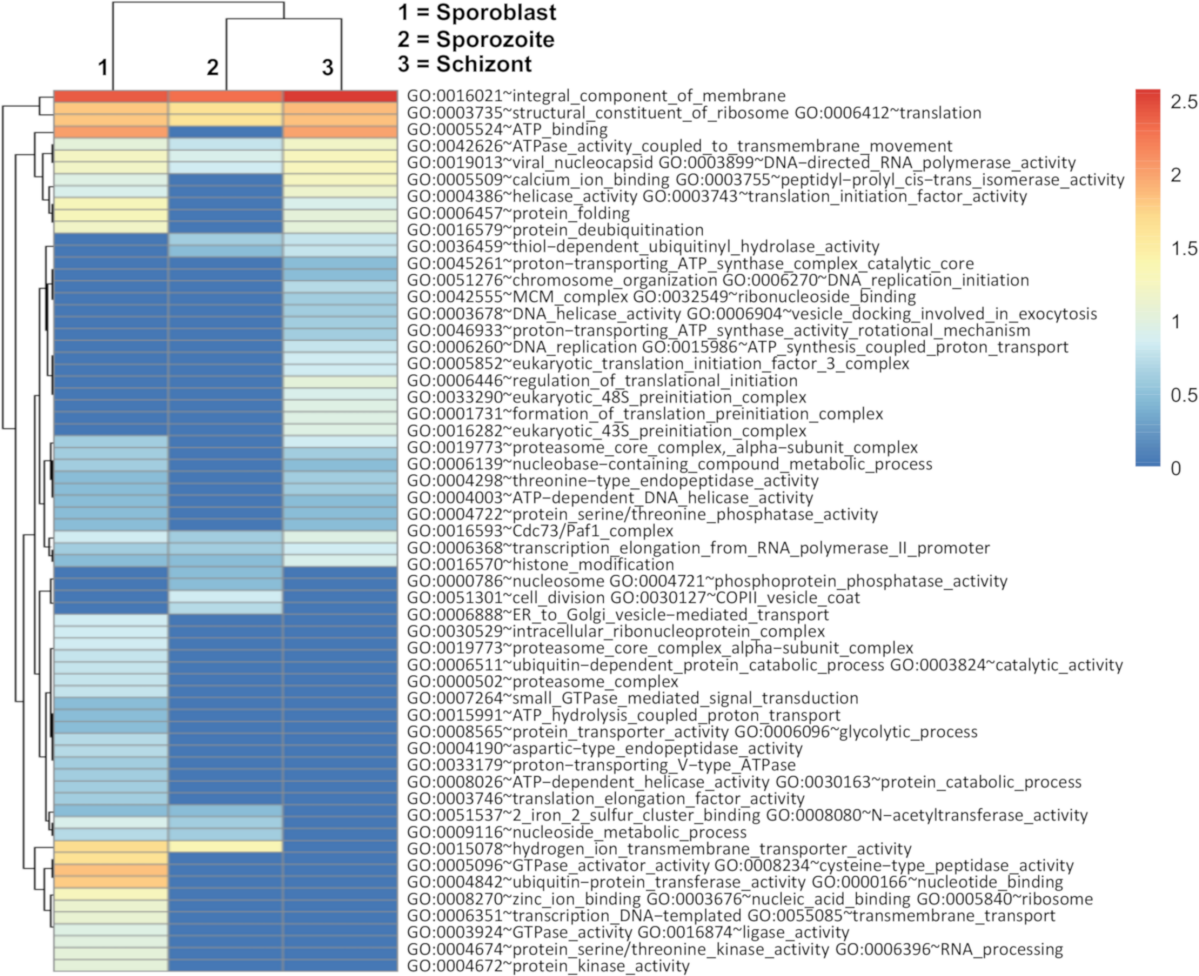
Heatmap showing the log_10_ (TPM values) for enriched GO terms enriched in the upregulated genes in the different infection stages obtained from DAVID.

We submitted the upregulated genes for each infection stage to DAVID for gene-annotation enrichment analysis. Genes with the same associated Gene Ontology (GO) term were clustered [27]. A high stringency cut-off was used to filter the clusters and the counts (number of genes with the specific associated GO term) retrieved. The GO categories that were most broadly enriched included ribosomal proteins and those involved in translation and integral components of the plasma membrane; ATP-binding was also enriched but only in sporozoite and schizont (Fig 6). In the sporozoite, we saw an enrichment in genes involved in zinc ion and nucleic acid binding, ribosomal activity, transcription, transmembrane transport, GTPase, ligase and protein kinase activities, glycolytic process, RNA processing, protease activity and translation elongation. Functions only enriched during sporoblast included vesicle-mediated transport (ER to Golgi), intracellular ribonucleoprotein complex and proteasome activity. Genes upregulated during sporozoite were mainly involved in zinc-ion and nucleic acid binding, ribosome and transcription. In the schizont stage, we see GO term enrichment for some genes with activities mainly in translation, DNA replication and cell division.

**Fig 7:**
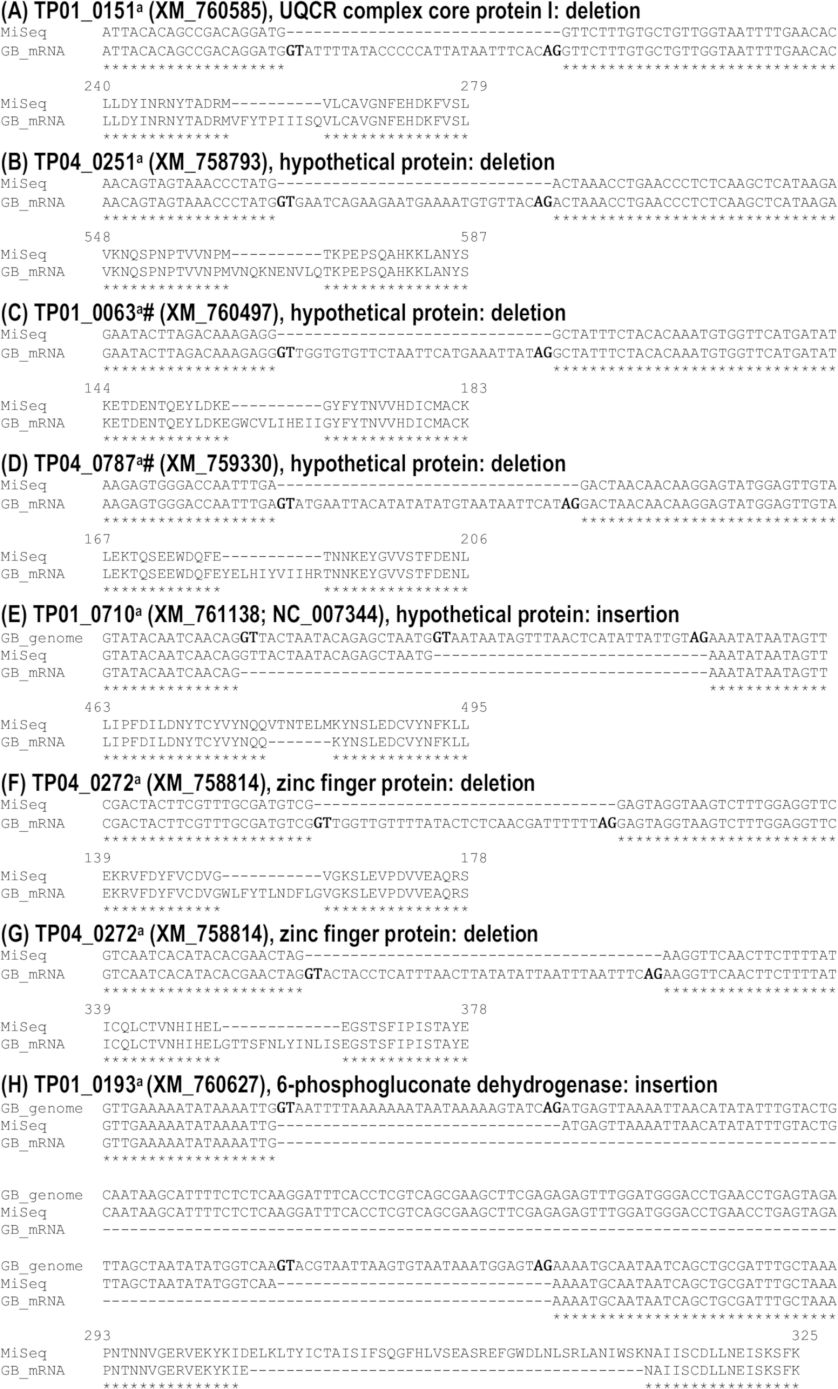
Indels identified in the transcripts. Multiple nucleotide and amino acid sequences alignments of *T*. *parva* gene regions with indels using the GT-AG dinucleotides DNA sequence requirement at the first two (GT) and last two (AG) positions of introns in pre-mRNAs (highlighted in bold). (^a^) indicates difference in both original genome annotation and re-annotation; # indicates introns not in-frame.

**Fig 8:**
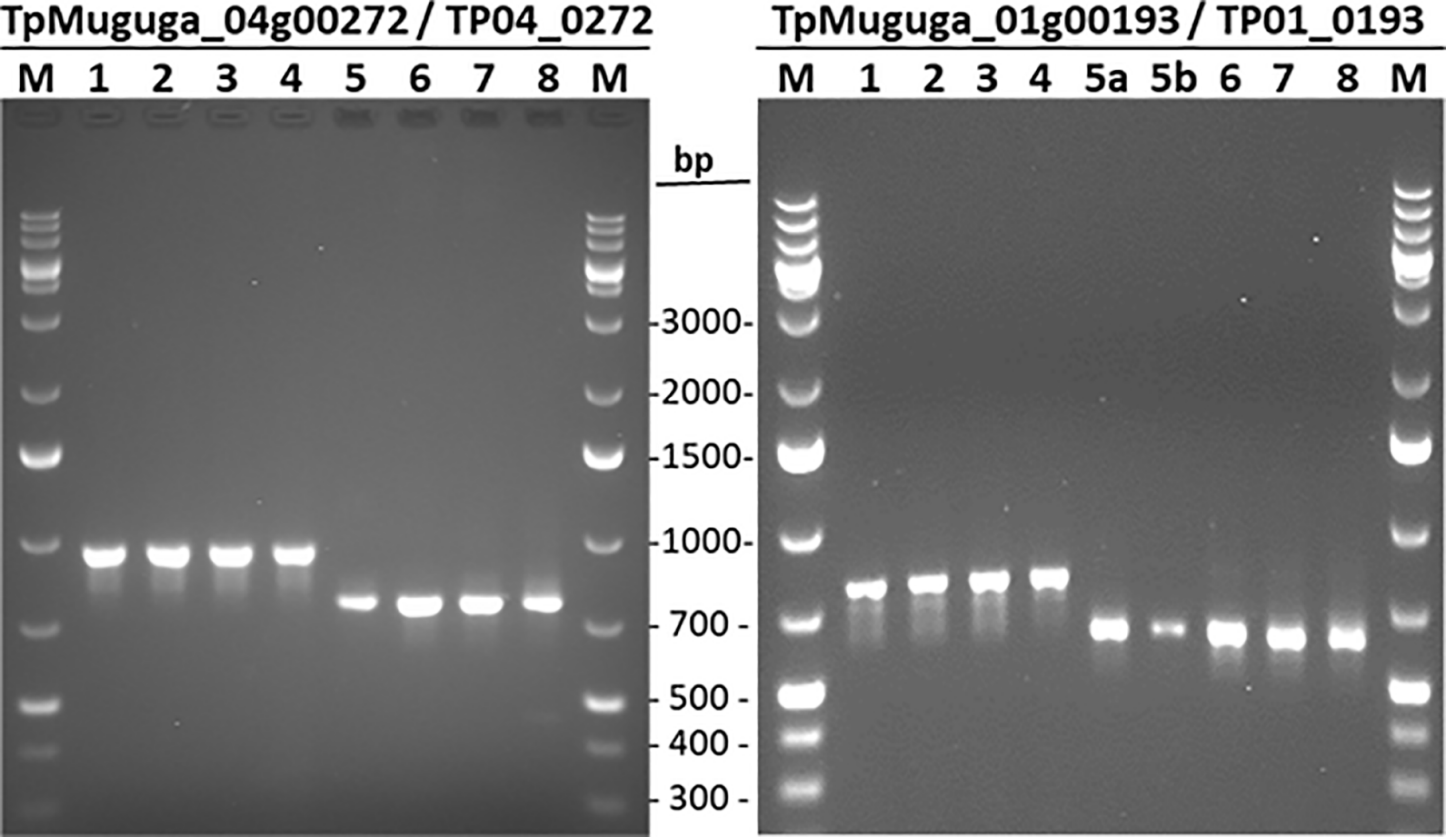
PCR verification of deletion (TP04_0272) and insertion (TP01_0193) identified by MiSeq. Samples are as follows: Lanes 1–4, genomic DNA from *T*. *parva* strain Muguga 3087 (lane 1); *T*. *parva* strain Marikebuni 3292 (lane 2); *T*. *parva* strain Kiambu5 (lane 3); *T*. *parva* F100 TpM (lane 4); Lanes 5–8, mRNA from sporozoite of *T*. *parva* Muguga 3087 (lanes 5, 5a and 5b); mRNA schizont *T*. *parva* Muguga 3087 (lane 6); schizont of *T*. *parva* Muguga 3087 (lane 7); piroplasm of *T*. *parva* Muguga 3087 (lane 8). Also shown are DNA size markers (lanes M), with length in base pair (pb).

This study found no variations in the sequence of transcripts throughout the three life cycle stages analysed and, in general, our data confirmed the recent reannotation of the *T*. *parva* genome (Tretina et al., in preparation). Nevertheless, we discovered some transcripts that were different in size from those in the original 2005 genome annotation and/or the re-annotation (Fig 7). The difference was due to indels, which we confirmed by gel analysis of PCR products (Fig 8) and Sanger sequencing of amplicons (Supplementary S2 File) for two genes. Thus, using gene-specific primers, the DNA region amplified from TpMuguga_01g00193 mRNA was 129 bp longer, whereas the one from TpMuguga_04g00272 mRNA was 72 bp shorter, than their respective sequences in the original and new annotations. We also observed that TP04_0251 was shorter in the re-annotation than in the original genome annotation and had been renamed TpMuguga_04g02465. All these genes have now been corrected in the new version of the re-annotation.

### Antigen expression is not conserved across the three infection stages

**Fig 9:**
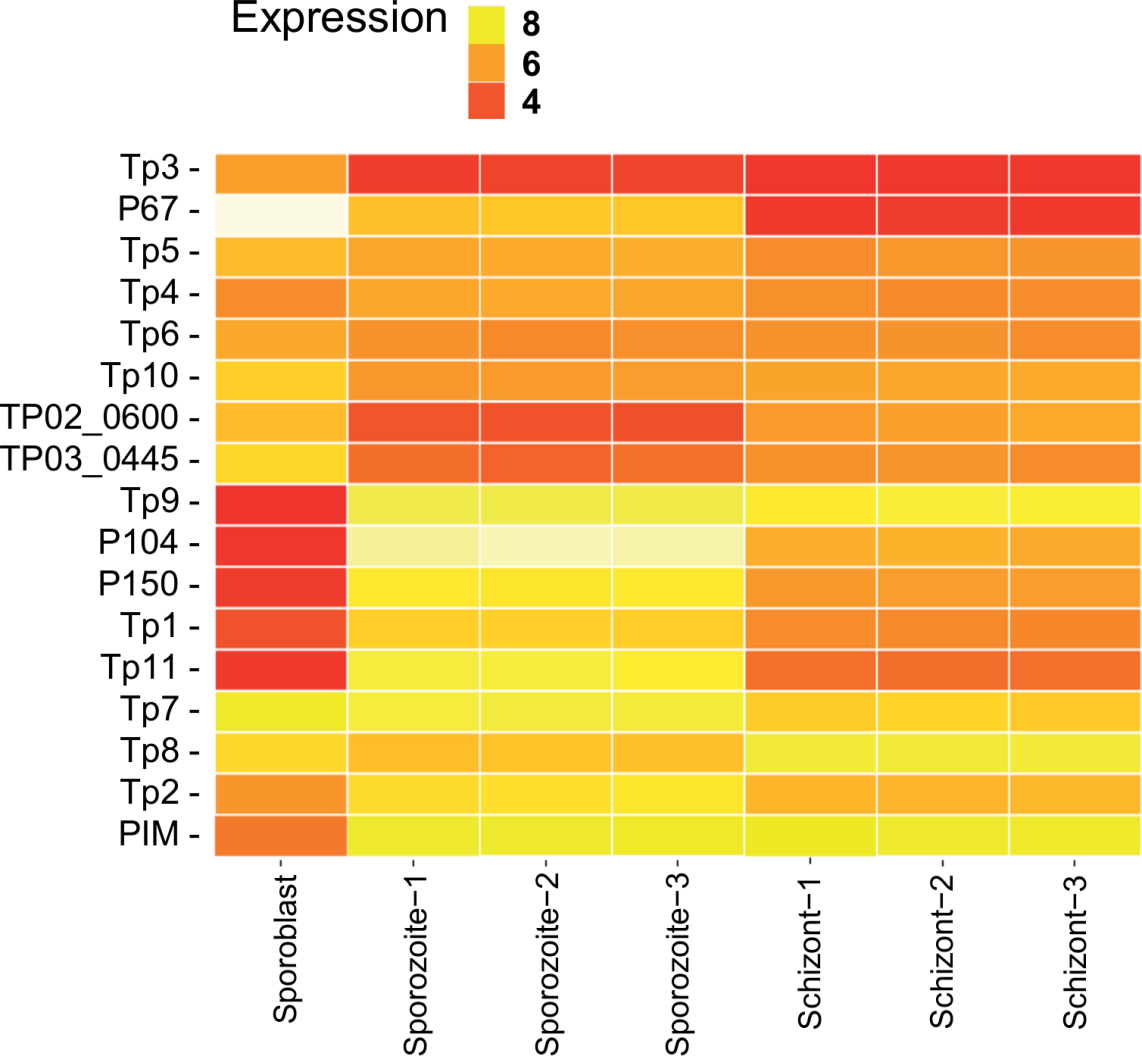
Heatmap showing the log_10_ of normalized TPM values for known *Theileria parva* antigens in samples from three parasite life cycle stages. Yellowish is high expression and red is low expression. Selected known candidate vaccine antigens are listed.

Several antigens have been characterized in *T*. *parva* [28, 29]. The antigens with low expression during the sporoblast stage (Tp2, Tp9, p104, p150 and PIM) are activated during the sporozoite stage (Fig 9). On the other hand, the antigens with a higher expression in sporoblast, such as p67 and the proliferating cell nuclear antigens TP02_0600 and TP03_0445, are downregulated during sporozoite although their expression increases again slightly in the schizont. Tp7 and Tp8 are highly and constantly expressed during all three life cycle stages. In general, most of the antigens have reduced expression during the schizont stage. Tp8, Tp9 and PIM are the only exceptions, with expression maintained or increased in that stage.

**Fig 10:**
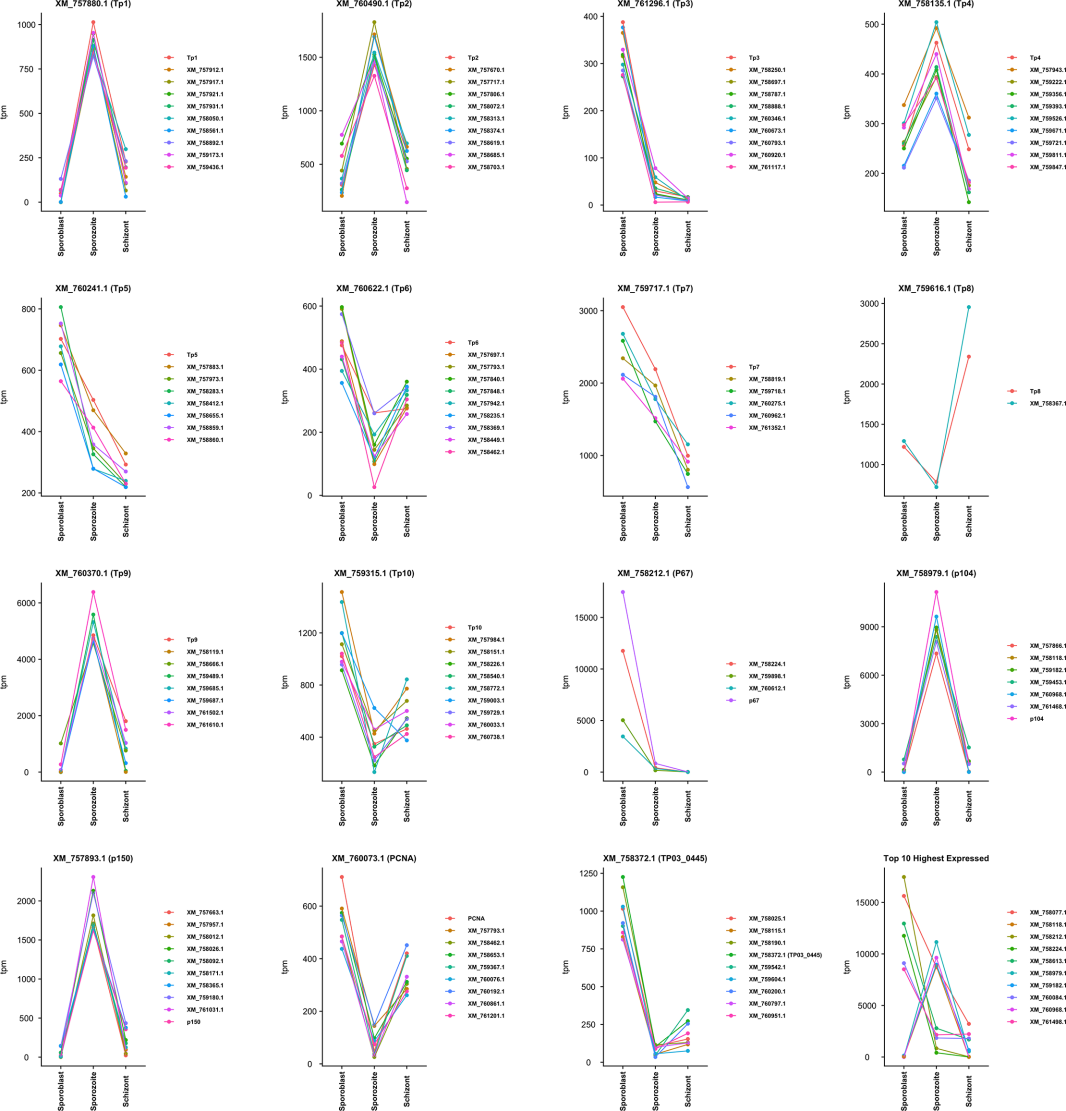
Clusters of expression (TPM) for known *Theileria parva* antigens. The genes with similar expression to the known antigens are shown in each plot legend.

The R package Cluster was used to find genes with similar expression to the known antigens. To compare the three infection stages, TPM values were considered for each gene and the genes falling in the same cluster as the antigens were plot together. Up to seven most similar genes to the antigen in each cluster were considered in each case (Fig 10). The cluster information can be found in the supplementary S1 Table. Only a few genes with a similar expression to p67 could be identified (TP03_0299, TP02_0425 and to a lesser extent TP01_0178), probably due to the very high expression of p67 in the sporoblast (Fig 10); like p67, these three contain a signal peptide. It is worth mentioning that genes like TP01_0380, TP01_1225 and TP01_1227 have similar expression profile to the antigen p104 (TP04_0437) and also contain a signal peptide.

We searched for orthologs of genes known in other apicomplexans to encode proteins associated with sporozoite invasion organelles. Structural properties of proteins encoded by nine rhoptry-, five microneme- and one dense granules-associated genes of *T*. *parva* are summarized in the supplementary S1 Table. They are differentially expressed and at relatively very low or moderate levels, except for the dense granules gene (TP02_0607) and four rhoptry genes (TP01_0701, TP02_0645, TP03_0067 and TP03_0655), with expression higher than 1,000 TPM units in at least one of the three parasite life cycle stages.

## Discussions

The intracellular parasite *Theileria parva* infects and reversibly transforms the bovine lymphocytes [7], using strategies that help the parasite survive, establish itself and proliferate within the host [30, 31, 32]. Some of these strategies involve modulation of the host immune response as well as molecular mimicry through host-parasite interaction [6]. In the present study of gene expression profiling, we demonstrated reproducible performance in our assays and, as shown in Fig 2B, there was no significant difference between replicates for each life cycle stage. Because protein coding housekeeping genes routinely used as endogenous controls in quantification studies of mRNA transcripts were shown to vary considerably between different *T*. *parva* stocks [33], they were not used in this study. They include genes encoding glyceraldehyde-3-phosphate dehydrogenase (GAPDH), cytochrome b and fructose-2.6-biphosphate aldolase (F6P) proteins. We did not use β-actin gene as control because it may be highly demanded in actively dividing parasites like sporoblast and schizont for motility, structure and integrity whereas its demand may be low in the non-replicating cells such as the sporozoite. In this regard, our study showed that β-actin (TP02_0903) was more expressed in the replicative sporoblast and schizont forms (5974 and 2499 counts, respectively) than in the non-replicating sporozoite form (1114 counts). Lastly, by using Transcripts Per Kilobase Million (TPM) in our study, we normalized both for gene length and sequencing depth, which improved comparisons across genes and across samples. We observed that the parasite development from the sporoblast, its transformation into sporozoite and establishment in the host cells as schizont is accompanied by generalized increase of differential gene expression. Most of these changes in gene expression may be associated with the ability of the parasite to gain entry into the host, and its persistence as well as interference with the signal transduction pathways that govern important functions of the infected cell [34]. The drastic increase of the number of upregulated genes in the schizont stage of the parasite shown here could be related to their key role in promoting the establishment of the parasite in the host cytosol. Interestingly, the four genes not expressed in the schizont stage (Fig 5D) were upregulated in the sporozoite stage. These tick stage-specific genes may play a critical role in the survival of the parasite in the tick vector, and not in the parasite establishment in, and/or transformation of, the host cell.

In the present study, the sequence of vast majority of the parasite transcripts matched perfectly well with the genes in the re-annotated genome available in GenBank. The very few sequence differences observed were confirmed by gene fragment analysis and corroborated our results, hence validating the power of the approach we used and the high level of accuracy of our sequencing data. Moreover, multiple sequence alignments of the mRNA with the genomic DNA sequences showed that, in general, the observed indels conform to the eukaryotic canonical splice site composition, i.e., the GT-AG dinucleotides DNA sequence requirement at the first two and last two positions of introns in pre-mRNAs. These findings reveal a usefull approach to identify incorrect annotations, and resulted in the improvement of the *T*. *parva* genome annotation.

It is reported that p67 expression is stage-specific, and restricted to the sporozoite [29]. In our study, we found that p67 is also expressed in the schizont, although at a very low level (21 TPM units) as compared to sporozoite (840 TPM). Interestingly, we found that p67 is also expressed in the sporoblast and 20 times more than in the sporozoite. In addition, we found that the three gene transcripts TP03_0299, TP02_0425 and TP01_0178 (corresponding to proteins XM_758224.1, XM_759898.1 and XM_760612.1, respectively), had similar transcription expression profiles to p67, an antigen used as a subunit vaccine against ECF [35]. These three antigens also have a signal peptide (but lack a transmembrane domain) suggesting that they are directed to the secretory pathway from the parasite secreted where they could reside in parasite organelles such the endoplasmic reticulum or golgi, be inserted into parasite cellular membrane, or be secreted from the parasite. Furthermore, the XM_760612.1 protein has a nuclear localization signal, suggesting a possible interaction with the host cell nucleus [36]. The high level of expression of the three genes in the tick stages of the parasite make them potential candidate antigens for the development of a transmission blocking subunit vaccine. Several genes with similar expression to other known *T*. *parva* vaccine antigens were also identified, some containing transmembrane domains and/or a signal peptide. The expression profile of these selected genes could be further evaluated using quantitative reverse transcription PCR; they could also be evaluated further for their potential as vaccine antigens. Additionally, the genes with a nuclear localization signal can be explored further for their potential role in host cell transformation. The two proliferating cell nuclear antigens (PCNA) (TP02_0600 and TP03_0445), homologs of which are known to be abundant in proliferating cells [37], were also more highly transcribed in the schizont than in the sporozoite. They might also have a role in host cell transformation by interacting with host cell regulatory proteins.

Using protein prediction software, we examined critically the four most highly expressed genes encoding hypothetical proteins. TP04_0640 protein showed homology and structural similarity with meiotically up-regulated gene 84 protein (MUG84) [38], i.e. with two transmembrane domains, a small loop-like structure on the membrane and casein kinase II phosphorylation sites, suggesting that it is associated with organellar membrane like ER. MUG84 is also a putative phosphatidylinositol N-acetylglucosaminyltransferase subunit P (PIG-P) domain-containing protein. Therefore, TP04_0640 protein is likely to have similar function as MUG84, i.e.: being involved in meiotic events and GPI anchor biosynthesis. The strong homology to proteins in other piroplasms, including *Theileria*, *Babesia* and Cytauxzoon species, suggests that this is likely a conserved protein involved in cellular metabolic processes. Its high level of expression in the sporozoite suggest a potential important role for this protein in that stage and during infection. Therefore, TP04_0640 protein appears as a potential target for the development of anti *Theileria* drugs.

The presence of several post-translational modification sites and a cell attachment motif suggests that the TP03_0299 protein may be involved in parasite-bovine and parasite-tick protein-protein interactions. Considering that it can also be secreted by the parasite, as suggested by the presence of a signal peptide, the TP03_0299 protein could serve as a potential disease biomarker or protein therapeutic target. Moreover, the fact that it is expressed almost exclusively in the arthropod stages makes TP03_0299 a good candidate for the development of a transmission blocking subunit vaccine to prevent parasite transmission by the tick vector.

The numerous post-translational modification motifs, especially the leucine zipper pattern, present in the TP01_0541 protein, suggests that it might interact with other proteins, with DNA motifs that have a binding affinity for leucine zippers, or both. In that regard, TP01_0541 protein may be strongly involved in transcriptional gene regulation in the sporozoite stage in which its expression is very high and to which it is confined. In addition, our analyses suggest the TP01_0541 protein is a non-classical secreted protein. This structural characteristic and the high expression level in the sporozoite stage make TP01_0541 a potential target for antitherapeutic drugs, and/or another potential candidate antigen for the development of a transmission blocking subunit vaccine.

Regarding the TP03_0193, the protein contained several post-translational modification sites, an indication that it may be involved in protein-proteins interactions. In addition, the presence of a non-ordinary secondary structure and the high level of expression in the sporozoite strongly suggest that the TP03_0193 protein may play a crucial function, especially in that tick stage, such as protein stability, folding, function and recognition. In this regard, TP03_0193 would be a potential target for the development of antiparasitic drugs.

The restricted taxonomic distribution of three of these four hypothetical proteins makes them potentially very interesting. While the core gene set is fairly conserved among *Theileria* species, antigens tend to be considerably more species-specific [14, 39]. Alternatively, their taxonomic restriction could be explained by a role of these proteins in parasite-vector interactions, rather than in host-parasite processes. This is particularly the case of TpMuguga_03g00299, which is highly expressed in the sporoblast but not in the sporozoite or the schizont stages.

From the present study, the panel of contigs that were unassigned to any species during mapping will be explored further to discover eventual novel parasite genes and their functions. The fraction of reads corresponding to mitochondrial genes (cytochrome oxidase) will also be studied further to gain more insight into mtDNA variations in the evolution and adaptation of *T*. *parva* in infection.

In summary, RNASeq analysis of the three *T*. *parva* developmental stages has revealed a drastic increase in the number of upregulated genes as *T*. *parva* sporozoites infect bovine cells and differentiate into the schizont forms. In contrast, the highest expressed genes occur in the sporoblast stage. The information generated here will help in enhancing knowledge into the parasite genes involved in infection process and transformation of host cells and contribute to the identification of additional vaccine antigens for the control of the ECF disease.

## Conclusion

The work presented here is a representation of the first, albeit partial, analysis of the gene expression profiles of *T*. *parva* developmental stages from the sporoblast through the sporozoite to the schizont stages. From our study, we were able to identify genes that are differentially expressed across those parasite life cycle stages. In particular, we identified genes that have an expression profile and protein domains similar to those of known *T*. *parva* antigens, such as p67. They can be explored further for their potential as candidate vaccine antigens or their role in parasite development. Our results are also likely to provide insight into the fascinating phenomenon of reversible host cell transformation, that could be of great importance in the control of ECF and similar diseases.

## Supporting information

S1 File Differentially expressed genes of unmapped reads. (CSV)

S2 File Confirmation of indels in the transcripts by PCR and sequencing. (DOCX)

S1 Table Average Transcripts Per Kilobase Million (TPM) of genes, genes hit by unmapped reads and, T. parva orthologs of apicomplexan genes encoding proteins of sporozoite invasion organelles. (XLSX)

S2 Table Differentially expressed genes between the three stages studied. (XLSX)
