# Supplementary material for: Transcriptomics reveal potential vaccine antigens and a drastic increase of upregulated genes during Theileria parva development from arthropod to bovine infective stages
Source: PLoS One. 2018 Oct 10;13(10):e0204047. doi: 10.1371/journal.pone.0204047 (PMC6179218; doi:10.1371/journal.pone.0204047)
Supplement: S2 File — (DOCX) [file pone.0204047.s002.docx]

**S2 File**. Confirmation of indels in the transcripts by using PCR and Sanger sequencing.

**List of T. parva DNA and mRNA templates used for PCR and RT-PCR.**

| **Lane in Fig 6** | **Template origin** | **Strain** | **Parasite stage** |
| --- | --- | --- | --- |
| 1 | Genomic DNA | Muguga 3087 | Piroplasm |
| 2 | Genomic DNA | Marikebuni clone 3292 | Piroplasm |
| 3 | Genomic DNA | Kiambu5 | Piroplasm |
| 4 | Genomic DNA | F100 TpM Muguga | Piroplasm |
| 5a | mRNA | Muguga 3087 | Sporozoite |
| 5b | mRNA | Muguga 3087 | Schizont |
| 6 | mRNA | Muguga 3087 | Schizont |
| 7 | mRNA | Muguga 3087 | Schizont |
| 8 | mRNA | Muguga 3087 | Piroplasms |

1. **Sequence analysis of TP01_0193 mRNA (TpMuguga_01g00193)**

| **Primers** | **Sequence (highlighted in the sequences)** | **Tm (1.5mM MgCl2)** |
| --- | --- | --- |
| TP01-0193-Fwd | 5’-GCCGTGTGTTTCCTCAAATG-3’ | 63 |
| TP01-0193-Rev | 5’-GGTGATTTCCTAGCATATCTACTCTT-3’ | 62.9 |

**Size of the DNA fragments and corresponding sequences.**

TP01_0193genomic = 774 bp (confirmed by PCR and sequencing)

TP01_0193mRNA = 482 bp

TP01_0193MiSeq = 611 bp (confirmed by PCR and sequencing)

**Sequences.**

>TP01_0193genome*

**GCCGTGTGTTTCCTCAAATG**GAACAGGAAAATGGACTGTTCAGGACGCATTTGACAGAGCGGTCCCAGTGCCTTCCATAGCAATTTCTGTAGACATGAGGTGTTTCAGTAATTTATCACAACATTCAAACATCCCTAACACTAATAATGTTGGTGAAAGGGTTGAAAAATATAAAATTGGTAATTTTAAAAAAATAATAAAAAGTATCAGATGAGTTAAAATTAACATATATTTGTACTGCAATAAGCATTTTCTCTCAAGGATTTCACCTCGTCAGCGAAGCTTCGAGAGAGTTTGGATGGGACCTGAACCTGAGTAGATTAGCTAATATATGGTCAAGTACGTAATTAAGTGTAATAAATGGAGTAGAAAATGCAATAATCAGCTGCGATTTGCTAAATGAAATCTCAAAATCATTCAAAGAAACTGATCGAATGTTGCCATTTCACTCAAAGTATAAAAAATATTAGTAACATTCAATAGGTTTAACGGAATATTAAAAGATAGCATGAAAACTTGGAAAGATGTTGTAAAAAGGTGTTTGGAAAATGATATTCCAGTACCAGGAATCGCAACCAGCTTACAATATATACAAATTCTGTTCAATAAACATGTAATTGTGGATTTTATTTAATTTAATTACCTAGTTAATTAGTCAATTAATTAATTGGTTGGTTGTTGAAATGGTATAGGAAGGATACAATTTAATACAAGCACAAAGAGATTGTTTTGGATCGCATACTTTCAG**AAGAGTAGATATGCTAGGAAATCACC**

>**TP01_0193**mRNA**

**GCCGTGTGTTTCCTCAAATG**GAACAGGAAAATGGACTGTTCAGGACGCATTTGACAGAGCGGTCCCAGTGCCTTCCATAGCAATTTCTGTAGACATGAGGTGTTTCAGTAATTTATCACAACATTCAAACATCCCTAACACTAATAATGTTGGTGAAAGGGTTGAAAAATATAAAATTGAAAATGCAATAATCAGCTGCGATTTGCTAAATGAAATCTCAAAATCATTCAAAGAAACTGATCGAATGTTGCCATTTCACTCAAAGTTTAACGGAATATTAAAAGATAGCATGAAAACTTGGAAAGATGTTGTAAAAAGGTGTTTGGAAAATGATATTCCAGTACCAGGAATCGCAACCAGCTTACAATATATACAAATTCTGTTCAATAAACATGTAATTGAAGGATACAATTTAATACAAGCACAAAGAGATTGTTTTGGATCGCATACTTTCAG**AAGAGTAGATATGCTAGGAAATCACC**

>**TP01_0193**MiSeq*

**GCCGTGTGTTTCCTCAAATG**GAACAGGAAAATGGACTGTTCAGGACGCATTTGACAGAGCGGTCCCAGTGCCTTCCATAGCAATTTCTGTAGACATGAGGTGTTTCAGTAATTTATCACAACATTCAAACATCCCTAACACTAATAATGTTGGTGAAAGGGTTGAAAAATATAAAATTGATGAGTTAAAATTAACATATATTTGTACTGCAATAAGCATTTTCTCTCAAGGATTTCACCTCGTCAGCGAAGCTTCGAGAGAGTTTGGATGGGACCTGAACCTGAGTAGATTAGCTAATATATGGTCAAAAAATGCAATAATCAGCTGCGATTTGCTAAATGAAATCTCAAAATCATTCAAAGAAACTGATCGAATGTTGCCATTTCACTCAAAGTTTAACGGAATATTAAAAGATAGCATGAAAACTTGGAAAGATGTTGTAAAAAGGTGTTTGGAAAATGATATTCCAGTACCAGGAATCGCAACCAGCTTACAATATATACAAATTCTGTTCAATAAACA**TGTAAT**TGAAGGATACAATTTAATACAAGCACAAAGAGATTGTTTTGGATCGCATACTTTCAG**AAGAGTAGATATGCTAGGAAATCACC**

*Sequences from this study

**Sequences from the GenBank

**CLUSTAL multiple sequence alignment of the DNA fragments.**

TP01_0193Genome GCCGTGTGTTTCCTCAAATGGAACAGGAAAATGGACTGTTCAGGACGCATTTGACAGAGC

TP01_0193MiSeq GCCGTGTGTTTCCTCAAATGGAACAGGAAAATGGACTGTTCAGGACGCATTTGACAGAGC

TP01_0193mRNA GCCGTGTGTTTCCTCAAATGGAACAGGAAAATGGACTGTTCAGGACGCATTTGACAGAGC

************************************************************

TP01_0193Genome GGTCCCAGTGCCTTCCATAGCAATTTCTGTAGACATGAGGTGTTTCAGTAATTTATCACA

TP01_0193MiSeq GGTCCCAGTGCCTTCCATAGCAATTTCTGTAGACATGAGGTGTTTCAGTAATTTATCACA

TP01_0193mRNA GGTCCCAGTGCCTTCCATAGCAATTTCTGTAGACATGAGGTGTTTCAGTAATTTATCACA

************************************************************

TP01_0193Genome ACATTCAAACATCCCTAACACTAATAATGTTGGTGAAAGGGTTGAAAAATATAAAATTG**G**

TP01_0193MiSeq ACATTCAAACATCCCTAACACTAATAATGTTGGTGAAAGGGTTGAAAAATATAAAATTG-

TP01_0193mRNA ACATTCAAACATCCCTAACACTAATAATGTTGGTGAAAGGGTTGAAAAATATAAAATTG-

***********************************************************

TP01_0193Genome **T**AATTTTAAAAAAATAATAAAAAGTATC**AG**ATGAGTTAAAATTAACATATATTTGTACTG

TP01_0193MiSeq ------------------------------ATGAGTTAAAATTAACATATATTTGTACTG

TP01_0193mRNA ------------------------------------------------------------

TP01_0193Genome CAATAAGCATTTTCTCTCAAGGATTTCACCTCGTCAGCGAAGCTTCGAGAGAGTTTGGAT

TP01_0193MiSeq CAATAAGCATTTTCTCTCAAGGATTTCACCTCGTCAGCGAAGCTTCGAGAGAGTTTGGAT

TP01_0193mRNA ------------------------------------------------------------

TP01_0193Genome GGGACCTGAACCTGAGTAGATTAGCTAATATATGGTCAA**GT**ACGTAATTAAGTGTAATAA

TP01_0193MiSeq GGGACCTGAACCTGAGTAGATTAGCTAATATATGGTCAA---------------------

TP01_0193mRNA ------------------------------------------------------------

TP01_0193Genome ATGGAGT**AG**AAAATGCAATAATCAGCTGCGATTTGCTAAATGAAATCTCAAAATCATTCA

TP01_0193MiSeq ---------AAAATGCAATAATCAGCTGCGATTTGCTAAATGAAATCTCAAAATCATTCA

TP01_0193mRNA ---------AAAATGCAATAATCAGCTGCGATTTGCTAAATGAAATCTCAAAATCATTCA

***************************************************

TP01_0193Genome AAGAAACTGATCGAATGTTGCCATTTCACTCAAA**GT**ATAAAAAATATTAGTAACATTCAA

TP01_0193MiSeq AAGAAACTGATCGAATGTTGCCATTTCACTCAAA--------------------------

TP01_0193mRNA AAGAAACTGATCGAATGTTGCCATTTCACTCAAA--------------------------

**********************************

TP01_0193Genome T**AG**GTTTAACGGAATATTAAAAGATAGCATGAAAACTTGGAAAGATGTTGTAAAAAGGTG

TP01_0193MiSeq ---GTTTAACGGAATATTAAAAGATAGCATGAAAACTTGGAAAGATGTTGTAAAAAGGTG

TP01_0193mRNA ---GTTTAACGGAATATTAAAAGATAGCATGAAAACTTGGAAAGATGTTGTAAAAAGGTG

*********************************************************

TP01_0193Genome TTTGGAAAATGATATTCCAGTACCAGGAATCGCAACCAGCTTACAATATATACAAATTCT

TP01_0193MiSeq TTTGGAAAATGATATTCCAGTACCAGGAATCGCAACCAGCTTACAATATATACAAATTCT

TP01_0193mRNA TTTGGAAAATGATATTCCAGTACCAGGAATCGCAACCAGCTTACAATATATACAAATTCT

************************************************************

TP01_0193Genome GTTCAATAAACATGTAATT**GT**GGATTTTATTTAATTTAATTACCTAGTTAATTAGTCAAT

TP01_0193MiSeq GTTCAATAAACATGTAATT-----------------------------------------

TP01_0193mRNA GTTCAATAAACATGTAATT-----------------------------------------

*******************

TP01_0193Genome TAATTAATTGGTTGGTTGTTGAAATGGTAT**AG**GAAGGATACAATTTAATACAAGCACAAA

TP01_0193MiSeq --------------------------------GAAGGATACAATTTAATACAAGCACAAA

TP01_0193mRNA --------------------------------GAAGGATACAATTTAATACAAGCACAAA

****************************

TP01_0193Genome GAGATTGTTTTGGATCGCATACTTTCAGAAGAGTAGATATGCTAGGAAATCACC

TP01_0193MiSeq GAGATTGTTTTGGATCGCATACTTTCAGAAGAGTAGATATGCTAGGAAATCACC

TP01_0193mRNA GAGATTGTTTTGGATCGCATACTTTCAGAAGAGTAGATATGCTAGGAAATCACC

******************************************************

**CLUSTAL multiple sequence alignment of the predicted amino acid sequences.**

TP01_0193mRNA** PCVSSNGTGKWTVQDAFDRAVPVPSIAISVDMRCFSNLSQHSNIPNTNNVGERVEKYKIE

TP01_0193MiSeq* PCVSSNGTGKWTVQDAFDRAVPVPSIAISVDMRCFSNLSQHSNIPNTNNVGERVEKYKID

***********************************************************:

TP01_0193mRNA** -------------------------------------------NAIISCDLLNEISKSFK

TP01_0193MiSeq* ELKLTYICTAISIFSQGFHLVSEASREFGWDLNLSRLANIWSKNAIISCDLLNEISKSFK

*****************

TP01_0193mRNA** ETDRMLPFHSKFNGILKDSMKTWKDVVKRCLENDIPVPGIATSLQYIQILFNKHVIEGYN

TP01_0193MiSeq* ETDRMLPFHSKFNGILKDSMKTWKDVVKRCLENDIPVPGIATSLQYIQILFNKHVIEGYN

************************************************************

TP01_0193mRNA** LIQAQRDCFGSHTFRRVDMLGNH

TP01_0193MiSeq* LIQAQRDCFGSHTFRRVDMLGNH

***********************

1. **Sequence analysis of TP04_0272 mRNA (TpMuguga_04g00272)**

| **Primer name** | **Sequence (highlighted in the sequences)** | **Tm (1.5 mM MgCl_2_)** |
| --- | --- | --- |
| **TP04_0272-Fwd** | GAACGGTAACCCATTGCCTTC | 64^o^C |
| **TP04_0272-Rev** | GTTGGTTCTTCCTCTCCCTC | 62^o^C |

**Size of the DNA fragments.**

TP04_0272genome = 966 bp (confirmed by PCR and sequencing)

TP04_0272mRNA = 839 bp

TP04_0272MiSeq = 767 bp (confirmed by PCR and sequencing)

**Sequences.**

>TP04_0272genome*

**GAACGGTAACCCATTGCCTTC**TAAAGCTCCTTCTGATTTTACCTCTTCAATTTATGGCGAGAAGAGGGTTTTCGACTACTTCGTTTGCGATGTCGGTTGGTTGTTTTATACTCTCAACGATTTTTTAGGAGTAGGTAAGTCTTTGGAGGTTCCTGACGTAGTTGAGGCTCAAAGATCCAGAGTATCGATGCCCGTAGAATATGACTCTTACTTTCTGACAAATTCAGAAAATTTGAGACTTGATTCTTTAACAGTTTTTGCTGGATCAAATGAGACCGAAAATGGCTCCGTGGAACTTTCTAGTGAAGCTCAATCCAAGGGTGTTTTACCCCAAAACACCTTCAAGCATAATTACATAATTTACGACTCATCACAGGTATTAACAAAAACATATTATTCAGACTAAATTACAAATTGTCAACTGCTTAATTGAGTCTTTAGATTTTACCGAGATATTTAATTCAATTTGAGTTTGACCCTAGTGCTGATGAGTCTTTTGCACTTCCTCTCTGCGATAACTGTCAAAGTGATGTGTCCACAATATACTGTCCTTCAGATAGCGCAAGGTATGTTTTATTAAATTTTATTACTTTTAGGATTTGCTCCAAGTGTGATGTTCGTCTTCACTCTAACAATAAGGTTGTTTCACGGCACATTAGGGTCCCCTTAAGCGAGGTACTTTTTTATCAAATTAACATCCTTTTTAGATGCCTAGGCCTTACTCCAAGTGTAGAATTCACCAGACAAAGTCATATCATCTTTACTGTACTGTCTGTGAAACTCCAATTTGCCAACTTTGCACCGTCAATCACATACACGAACTAGGTACTACCTCATTTAACTTATATATTAATTTAATTTCAGAAGGTTCAACTTCTTTTATTCCTATTTCAACTGCTTATGAGGCTGTGGTTCAAAATTTAACCTCCACTACTGACTTTTCTTT**GAGGGAGAGGAAGAACCAAC**

>TP04_0272mRNA**

**GAACGGTAACCCATTGCCTTC**TAAAGCTCCTTCTGATTTTACCTCTTCAATTTATGGCGAGAAGAGGGTTTTCGACTACTTCGTTTGCGATGTCGGTTGGTTGTTTTATACTCTCAACGATTTTTTAGGAGTAGGTAAGTCTTTGGAGGTTCCTGACGTAGTTGAGGCTCAAAGATCCAGAGTATCGATGCCCGTAGAATATGACTCTTACTTTCTGACAAATTCAGAAAATTTGAGACTTGATTCTTTAACAGTTTTTGCTGGATCAAATGAGACCGAAAATGGCTCCGTGGAACTTTCTAGTGAAGCTCAATCCAAGGGTGTTTTACCCCAAAACACCTTCAAGCATAATTACATAATTTACGACTCATCACAGATTTTACCGAGATATTTAATTCAATTTGAGTTTGACCCTAGTGCTGATGAGTCTTTTGCACTTCCTCTCTGCGATAACTGTCAAAGTGATGTGTCCACAATATACTGTCCTTCAGATAGCGCAAGGATTTGCTCCAAGTGTGATGTTCGTCTTCACTCTAACAATAAGGTTGTTTCACGGCACATTAGGGTCCCCTTAAGCGAGATGCCTAGGCCTTACTCCAAGTGTAGAATTCACCAGACAAAGTCATATCATCTTTACTGTACTGTCTGTGAAACTCCAATTTGCCAACTTTGCACCGTCAATCACATACACGAACTAGGTACTACCTCATTTAACTTATATATTAATTTAATTTCAGAAGGTTCAACTTCTTTTATTCCTATTTCAACTGCTTATGAGGCTGTGGTTCAAAATTTAACCTCCACTACTGACTTTTCTTT**GAGGGAGAGGAAGAACCAAC**

>TP04_0272MiSeq*

**GAACGGTAACCCATTGCCTTC**TAAAGCTCCTTCTGATTTTACCTCTTCAATTTATGGCGAGAAGAGGGTTTTCGACTACTTCGTTTGCGATGTCGGAGTAGGTAAGTCTTTGGAGGTTCCTGACGTAGTTGAGGCTCAAAGATCCAGAGTATCGATGCCCGTAGAATATGACTCTTACTTTCTGACAAATTCAGAAAATTTGAGACTTGATTCTTTAACAGTTTTTGCTGGATCAAATGAGACCGAAAATGGCTCCGTGGAACTTTCTAGTGAAGCTCAATCCAAGGGTGTTTTACCCCAAAACACCTTCAAGCATAATTACATAATTTACGACTCATCACAGATTTTACCGAGATATTTAATTCAATTTGAGTTTGACCCTAGTGCTGATGAGTCTTTTGCACTTCCTCTCTGCGATAACTGTCAAAGTGATGTGTCCACAATATACTGTCCTTCAGATAGCGCAAGGATTTGCTCCAAGTGTGATGTTCGTCTTCACTCTAACAATAAGGTTGTTTCACGGCACATTAGGGTCCCCTTAAGCGAGATGCCTAGGCCTTACTCCAAGTGTAGAATTCACCAGACAAAGTCATATCATCTTTACTGTACTGTCTGTGAAACTCCAATTTGCCAACTTTGCACCGTCAATCACATACACGAACTAGAAGGTTCAACTTCTTTTATTCCTATTTCAACTGCTTATGAGGCTGTGGTTCAAAATTTAACCTCCACTACTGACTTTTCTTT**GAGGGAGAGGAAGAACCAAC**

*Sequences from this study

**Sequences from the GenBank

**CLUSTAL multiple sequence alignment of the DNA sequences.**

TP04_0272genome GAACGGTAACCCATTGCCTTCTAAAGCTCCTTCTGATTTTACCTCTTCAATTTATGGCGA

TP04_0272MiSeq GAACGGTAACCCATTGCCTTCTAAAGCTCCTTCTGATTTTACCTCTTCAATTTATGGCGA

TP04_0272mRNA GAACGGTAACCCATTGCCTTCTAAAGCTCCTTCTGATTTTACCTCTTCAATTTATGGCGA

************************************************************

TP04_0272genome GAAGAGGGTTTTCGACTACTTCGTTTGCGATGTCG**GT**TGGTTGTTTTATACTCTCAACGA

TP04_0272MiSeq GAAGAGGGTTTTCGACTACTTCGTTTGCGATGTCG-------------------------

TP04_0272mRNA GAAGAGGGTTTTCGACTACTTCGTTTGCGATGTCGGTTGGTTGTTTTATACTCTCAACGA

***********************************

TP04_0272genome TTTTTT**AG**GAGTAGGTAAGTCTTTGGAGGTTCCTGACGTAGTTGAGGCTCAAAGATCCAG

TP04_0272MiSeq --------GAGTAGGTAAGTCTTTGGAGGTTCCTGACGTAGTTGAGGCTCAAAGATCCAG

TP04_0272mRNA TTTTTTAGGAGTAGGTAAGTCTTTGGAGGTTCCTGACGTAGTTGAGGCTCAAAGATCCAG

****************************************************

TP04_0272genome AGTATCGATGCCCGTAGAATATGACTCTTACTTTCTGACAAATTCAGAAAATTTGAGACT

TP04_0272MiSeq AGTATCGATGCCCGTAGAATATGACTCTTACTTTCTGACAAATTCAGAAAATTTGAGACT

TP04_0272mRNA AGTATCGATGCCCGTAGAATATGACTCTTACTTTCTGACAAATTCAGAAAATTTGAGACT

************************************************************

TP04_0272genome TGATTCTTTAACAGTTTTTGCTGGATCAAATGAGACCGAAAATGGCTCCGTGGAACTTTC

TP04_0272MiSeq TGATTCTTTAACAGTTTTTGCTGGATCAAATGAGACCGAAAATGGCTCCGTGGAACTTTC

TP04_0272mRNA TGATTCTTTAACAGTTTTTGCTGGATCAAATGAGACCGAAAATGGCTCCGTGGAACTTTC

************************************************************

TP04_0272genome TAGTGAAGCTCAATCCAAGGGTGTTTTACCCCAAAACACCTTCAAGCATAATTACATAAT

TP04_0272MiSeq TAGTGAAGCTCAATCCAAGGGTGTTTTACCCCAAAACACCTTCAAGCATAATTACATAAT

TP04_0272mRNA TAGTGAAGCTCAATCCAAGGGTGTTTTACCCCAAAACACCTTCAAGCATAATTACATAAT

************************************************************

TP04_0272genome TTACGACTCATCACAG**GT**ATTAACAAAAACATATTATTCAGACTAAATTACAAATTGTCA

TP04_0272MiSeq TTACGACTCATCACAG--------------------------------------------

TP04_0272mRNA TTACGACTCATCACAG--------------------------------------------

****************

TP04_0272genome ACTGCTTAATTGAGTCTTT**AG**ATTTTACCGAGATATTTAATTCAATTTGAGTTTGACCCT

TP04_0272MiSeq ---------------------ATTTTACCGAGATATTTAATTCAATTTGAGTTTGACCCT

TP04_0272mRNA ---------------------ATTTTACCGAGATATTTAATTCAATTTGAGTTTGACCCT

***************************************

TP04_0272genome AGTGCTGATGAGTCTTTTGCACTTCCTCTCTGCGATAACTGTCAAAGTGATGTGTCCACA

TP04_0272MiSeq AGTGCTGATGAGTCTTTTGCACTTCCTCTCTGCGATAACTGTCAAAGTGATGTGTCCACA

TP04_0272mRNA AGTGCTGATGAGTCTTTTGCACTTCCTCTCTGCGATAACTGTCAAAGTGATGTGTCCACA

************************************************************

TP04_0272genome ATATACTGTCCTTCAGATAGCGCAAGGTATGTTTTATTAAATTTTATTACTTTTAGGATT

TP04_0272MiSeq ATATACTGTCCTTCAGATAGCGCAAG------------------------------GATT

TP04_0272mRNA ATATACTGTCCTTCAGATAGCGCAAG------------------------------GATT

************************** ****

TP04_0272genome TGCTCCAAGTGTGATGTTCGTCTTCACTCTAACAATAAGGTTGTTTCACGGCACATTAGG

TP04_0272MiSeq TGCTCCAAGTGTGATGTTCGTCTTCACTCTAACAATAAGGTTGTTTCACGGCACATTAGG

TP04_0272mRNA TGCTCCAAGTGTGATGTTCGTCTTCACTCTAACAATAAGGTTGTTTCACGGCACATTAGG

************************************************************

TP04_0272genome GTCCCCTTAAGCGAGGTACTTTTTTATCAAATTAACATCCTTTTTAGATGCCTAGGCCTT

TP04_0272MiSeq GTCCCCTTAAGCGAG--------------------------------ATGCCTAGGCCTT

TP04_0272mRNA GTCCCCTTAAGCGAG--------------------------------ATGCCTAGGCCTT

*************** *************

TP04_0272genome ACTCCAAGTGTAGAATTCACCAGACAAAGTCATATCATCTTTACTGTACTGTCTGTGAAA

TP04_0272MiSeq ACTCCAAGTGTAGAATTCACCAGACAAAGTCATATCATCTTTACTGTACTGTCTGTGAAA

TP04_0272mRNA ACTCCAAGTGTAGAATTCACCAGACAAAGTCATATCATCTTTACTGTACTGTCTGTGAAA

************************************************************

TP04_0272genome CTCCAATTTGCCAACTTTGCACCGTCAATCACATACACGAACTAG**GT**ACTACCTCATTTA

TP04_0272MiSeq CTCCAATTTGCCAACTTTGCACCGTCAATCACATACACGAACTAG---------------

TP04_0272mRNA CTCCAATTTGCCAACTTTGCACCGTCAATCACATACACGAACTAGGTACTACCTCATTTA

*********************************************

TP04_0272genome ACTTATATATTAATTTAATTTC**AG**AAGGTTCAACTTCTTTTATTCCTATTTCAACTGCTT

TP04_0272MiSeq ------------------------AAGGTTCAACTTCTTTTATTCCTATTTCAACTGCTT

TP04_0272mRNA ACTTATATATTAATTTAATTTCAGAAGGTTCAACTTCTTTTATTCCTATTTCAACTGCTT

************************************

TP04_0272genome ATGAGGCTGTGGTTCAAAATTTAACCTCCACTACTGACTTTTCTTTGAGGGAGAGGAAGA

TP04_0272MiSeq ATGAGGCTGTGGTTCAAAATTTAACCTCCACTACTGACTTTTCTTTGAGGGAGAGGAAGA

TP04_0272mRNA ATGAGGCTGTGGTTCAAAATTTAACCTCCACTACTGACTTTTCTTTGAGGGAGAGGAAGA

************************************************************

TP04_0272genome ACCAAC

TP04_0272MiSeq ACCAAC

TP04_0272mRNA ACCAAC

******

**CLUSTAL multiple sequence alignment of predicted amino acid sequences.**

TP04_0272mRNA** NGNPLPSKAPSDFTSSIYGEKRVFDYFVCDVGWLFYTLNDFLGVGKSLEVPDVVEAQRSR

TP04_0272MiSeq* NGNPLPSKAPSDFTSSIYGEKRVFDYFVCDVG-----------VGKSLEVPDVVEAQRSR

******************************** *****************

TP04_0272mRNA** VSMPVEYDSYFLTNSENLRLDSLTVFAGSNETENGSVELSSEAQSKGVLPQNTFKHNYII

TP04_0272MiSeq* VSMPVEYDSYFLTNSENLRLDSLTVFAGSNETENGSVELSSEAQSKGVLPQNTFKHNYII

************************************************************

TP04_0272mRNA** YDSSQILPRYLIQFEFDPSADESFALPLCDNCQSDVSTIYCPSDSARICSKCDVRLHSNN

TP04_0272MiSeq* YDSSQILPRYLIQFEFDPSADESFALPLCDNCQSDVSTIYCPSDSARICSKCDVRLHSNN

************************************************************

TP04_0272mRNA** KVVSRHIRVPLSEMPRPYSKCRIHQTKSYHLYCTVCETPICQLCTVNHIHELGTTSFNLY

TP04_0272MiSeq* KVVSRHIRVPLSEMPRPYSKCRIHQTKSYHLYCTVCETPICQLCTVNHIHEL--------

****************************************************

TP04_0272mRNA** INLISEGSTSFIPISTAYEAVVQNLTSTTDFSLRERKNQ

TP04_0272MiSeq* -----EGSTSFIPISTAYEAVVQNLTSTTDFSLRERKNQ

**********************************
